# Comprehensive Biotransformation Analysis of Phenylalanine-Tyrosine Metabolism Reveals Alternative Routes of Metabolite Clearance in Nitisinone-Treated Alkaptonuria

**DOI:** 10.3390/metabo12100927

**Published:** 2022-09-29

**Authors:** Brendan P. Norman, Andrew S. Davison, Bryony Hickton, Gordon A. Ross, Anna M. Milan, Andrew T. Hughes, Peter J. M. Wilson, Hazel Sutherland, Juliette H. Hughes, Norman B. Roberts, George Bou-Gharios, James A. Gallagher, Lakshminarayan R. Ranganath

**Affiliations:** 1 Department of Musculoskeletal and Ageing Science, Institute of Life Course and Medical Sciences, University of Liverpool, Liverpool L7 8TX, UK; 2 Liverpool Clinical Laboratories, Department of Clinical Biochemistry and Metabolic Medicine, Liverpool University Hospitals NHS Foundation Trust, Liverpool L69 3GA, UK; 3 Agilent Technologies UK Ltd., Cheadle SK8 3GR, UK; 4 Research Institute for Sport and Exercise Sciences, Liverpool John Moores University, Liverpool L3 3AF, UK; 5 Faculty of Health Social Care & Medicine, Edge Hill University, Ormskirk L39 4QP, UK

**Keywords:** alkaptonuria, hypertyrosinaemia, biotransformations, detoxification, metabolomics

## Abstract

Metabolomic analyses in alkaptonuria (AKU) have recently revealed alternative pathways in phenylalanine-tyrosine (phe-tyr) metabolism from biotransformation of homogentisic acid (HGA), the active molecule in this disease. The aim of this research was to study the phe-tyr metabolic pathway and whether the metabolites upstream of HGA, increased in nitisinone-treated patients, also undergo phase 1 and 2 biotransformation reactions. Metabolomic analyses were performed on serum and urine from patients partaking in the SONIA 2 phase 3 international randomised-controlled trial of nitisinone in AKU (EudraCT no. 2013-001633-41). Serum and urine samples were taken from the same patients at baseline (pre-nitisinone) then at 24 and 48 months on nitisinone treatment (patients N = 47 serum; 53 urine) or no treatment (patients N = 45 serum; 50 urine). Targeted feature extraction was performed to specifically mine data for the entire complement of theoretically predicted phase 1 and 2 biotransformation products derived from phenylalanine, tyrosine, 4-hydroxyphenylpyruvic acid and 4-hydroxyphenyllactic acid, in addition to phenylalanine-derived metabolites with known increases in phenylketonuria. In total, we observed 13 phase 1 and 2 biotransformation products from phenylalanine through to HGA. Each of these products were observed in urine and two were detected in serum. The derivatives of the metabolites upstream of HGA were markedly increased in urine of nitisinone-treated patients (fold change 1.2–16.2) and increases in 12 of these compounds were directly proportional to the degree of nitisinone-induced hypertyrosinaemia (correlation coefficient with serum tyrosine = 0.2–0.7). Increases in the urinary phenylalanine metabolites were also observed across consecutive visits in the treated group. Nitisinone treatment results in marked increases in a wider network of phe-tyr metabolites than shown before. This network comprises alternative biotransformation products from the major metabolites of this pathway, produced by reactions including hydration (phase 1) and bioconjugation (phase 2) of acetyl, methyl, acetylcysteine, glucuronide, glycine and sulfate groups. We propose that these alternative routes of phe-tyr metabolism, predominantly in urine, minimise tyrosinaemia as well as phenylalanaemia.

## 1. Introduction

The phenylalanine-tyrosine (phe-tyr) catabolic pathway is the major degradation route of the amino acids phenylalanine and tyrosine not required for protein synthesis. Phenylalanine and tyrosine are typically consumed in excess of daily nutritional requirements in adequately nourished populations and the metabolism of these amino acids results in conversion to fumarate and acetoacetate via several intermediary steps [1].

Rare disorders caused by mutations in genes encoding enzymes of phe-tyr catabolism demonstrate the potentially serious consequences of alterations in this pathway. Among these conditions, phenylketonuria (PKU; OMIM #261600) is the most prevalent and widely recognised disorder of amino acid metabolism. Classic PKU is caused by defective phenylalanine hydroxylase, resulting in increased blood phenylalanine concentration and increased urine metabolites phenylpyruvate, phenyllactate and phenylacetate [2]. The most severe inherited disorder of phe-tyr metabolism is hereditary tyrosinaemia type 1 (HT-1; OMIM #276700), a lethal disorder in infancy if untreated due to accumulation of toxic metabolites fumarylacetoacetate, maleylacetoacetate and their derivates succinylacetoacetate and succinylacetone [3]. Alkaptonuria (AKU; OMIM #203500) is a progressive multi-system disease attributable to accumulation of the tyrosine metabolite homogentisic acid (HGA), the active molecule in this disease, which directly causes the dominant features of severe, early-onset osteoarthropathy and cardiac valve disease [4]. The orphan drug nitisinone, now the standard treatment for HT-1 and AKU, inhibits the enzyme hydroxphenylpyruvate dioxygenase (HPPD), preventing the formation of the downstream metabolites that are toxic in these conditions [4,5,6,7,8,9,10,11,12,13].

Traditionally, efforts to characterise the metabolic alterations in the inherited disorders of phe-tyr metabolism have focused solely on the established metabolites of the pathway. In AKU and HT-1, it is well established that nitisinone treatment results in the undesired effect of increased concentrations of upstream metabolites; most notably in circulating tyrosine, known as hypertyrosinaemia, but also in the metabolites 4-hydroxyphenylpyruvic acid (HPPA) and 4-hydroxyphenyllactic acid (HPLA) which are more proximal to HPPD. Nitisinone-induced hypertyrosinaemia is a serious concern, associated with corneal keratopathy, dermal toxicity, tyrosine accumulation in brain and dysregulation of wider metabolism [11,12,14,15,16,17,18,19,20,21,22].

Recently, significant advances have been made in understanding the wider metabolic consequences caused by genetic mutations in phe-tyr metabolism and treatment with nitisinone beyond the traditional pathway. Untargeted metabolomic approaches revealed an extensive pattern of alteration to serum and urine metabolite pathways in patients and mice on nitisinone, which extends far beyond immediate phe-tyr metabolism and into metabolic pathways including those of purine, tryptophan and the TCA cycle [15,17,19,22,23]. A metabolomic approach has also elucidated previously undescribed pathways derived from HGA, including phase 1 and 2 biotransformation reactions of HGA which appear to be active in untreated AKU for adaptive detoxification [23]. Biotransformations are reactions through which an endogenous or exogenous molecule is metabolised by one or more enzymes to a more hydrophilic moiety which is more easily eliminated [24].

Knowledge of the alternative pathways within phe-tyr metabolism has potential to inform the clinical management of such disorders. Measurement of total pathway flux including alternative metabolic routes is important for routine monitoring of the disease and treatment response; in all of these conditions, patients are currently required to limit dietary intake of phe and/or tyr to manage the disease and potential adverse effects of treatment. In this analysis we investigated whether previously unreported biotransformations exist throughout the phe-tyr pathway from phenylalanine to HGA based on serum and urine metabolomic data acquired as part of the SONIA 2 (Suitability of Nitisinone In Alkaptonuria) phase 3 international randomised controlled trial of nitisinone in AKU. SONIA 2 presents a unique opportunity to mine for theoretically possible, but unproven phase 1 and 2 biotransformation products in phe-tyr metabolism. Given the marked increases in metabolites proximal to the inhibition of HPPD by nitisinone, namely HPPA, HPLA, tyrosine and phenylalanine, we hypothesised that these metabolites undergo similar biotransformations to HGA in untreated AKU as a means of increasing excretion and thus preventing accumulation in patients on nitisinone. The four-year duration of SONIA 2 enabled us to study the long-term longitudinal profiles of such metabolites in response to nitisinone, complementing previous analyses of SONIA 2 data which employed targeted data acquisition and were therefore restricted to pre-specified metabolites of the traditional phe-tyr pathway [25]. 

## 2. Materials and Methods

### 2.1. Reagents

Deionised water for mobile phases was purified in-house by DIRECT-Q 3UV water purification system (Millipore, Watford, UK). Methanol, ethanol, isopropanol (Sigma–Aldrich, Poole, UK), formic acid (Biosolve, Valkenswaard, The Netherlands) and ammonium formate (Fisher Scientific, Schwerte, Germany) were LC/MS grade. Captiva EMR-lipid 96-well plates (Agilent, Cheadle, UK) were used for metabolite extractions on serum samples.

### 2.2. Study Design and Patients

Samples studied were from patients partaking in the SONIA 2 clinical trial of nitisinone in AKU. SONIA 2 was a 4-year, open-label, evaluator-blind, randomised, no treatment controlled, parallel-group study undertaken at three study sites; Liverpool (UK), Paris (France) and Piešťany (Slovakia). The details and outcomes of the trial are published [12]. Serum and urine samples were collected from participants at baseline (pre-treatment) then at 3 months and 1, 2, 3 and 4 years on 10 mg oral daily nitisinone (Orfadin^®,^ Swedish Orphan Biovitrum, Stockholm, Sweden) treatment or no treatment. The serum and urine samples from visit 1 (baseline; pre-treatment), visit 4 (2 years), visit 6 (4 years) underwent metabolomic analysis. In this study, only the data from patients with samples available for these three time points were included; 53 and 50 patients in treated and untreated groups, respectively, for urine, and 47 and 45 patients in treated and untreated groups, respectively, for serum. Demographic information on the patients studied in this analysis is provided in Table 1. There were no restrictions regarding concomitant medications and patients in treatment and control groups could freely use analgesics, anti-inflammatory drugs and others as needed to treat symptoms of AKU. Patient diet was not actively managed in SONIA 2, apart from providing information sheets regarding eating a lower-protein diet. 

Ethical approval for the study was obtained from each of the participant sites including the North West—Liverpool Central Research Ethics Committee (reference: 13/NW/0567), the NURCH Ethical Committee in Piešťany (reference number: 04196/0029/001/001) and the EC Ile De France II committee in Paris (reference number: 2013-08-08). Informed consent was obtained from all participants.

All sample analyses were performed at the Department of Clinical Biochemistry, Liverpool Clinical Laboratories, Liverpool University Hospital NHS Foundation Trust.

### 2.3. Sample Collection and Preparation

Serum and urine samples were collected at the three study sites; Liverpool, Paris and Piešťany. Samples collected at Paris and Piešťany were transported frozen to the Royal Liverpool University Hospital for metabolite analysis and storage at −80 °C.

Serum samples (S-monovette, Sarstedt, Germany) were collected from patients after an overnight fast (≥8 h). Samples were centrifuged at 1500× *g* for 10 min at 4 °C; and the supernatant was stored at −20 °C until analysis. Serum samples were prepared using the Agilent Bravo metabolomics platform. This automated platform was programmed to pipette 100 µL of serum into a 96-well plate from a 96-well plate that contained 200 µL of serum. Following this, 450 μL of a 1:1 mixture of methanol:ethanol was added to the plate to precipitate proteins. The supernatant was then applied to a Captiva EMR-lipid 96-well plate and left to stand for 5 min and a vacuum was applied to the plate (2–5 Hg) to initiate flow. Eluents were collected into 1 mL 96-well plates and subsequently dried under a stream of nitrogen. Prior to analysis, samples were reconstituted with 100 µL of 10% methanol and were agitated on a plate shaker (MTS 2/4m IKA) at 600 rpm for 10 min.

Urine was collected over 24 h into 2.5 L bottles containing 30 mL of 5 mol/L sulphuric acid which was subsequently aliquoted and stored at −80 °C. Prior to analysis, urine samples were thawed at room temperature before vortexing and centrifugation at 1500× *g* for 5 min. A total of 150 µL of each urine sample was aliquoted into a 1 mL 96-well plate (Waters Corporation, Wilmslow, UK) and diluted with 450 µL of deionised water. Samples were mixed on a plate shaker (MTS 2/4m IKA) at 600 rpm for 10 min and sub-aliquoted into multiple replicate 96-well plates before storage at −80 °C ready for analysis. Prior to analysis, sample plates were thawed then agitated on a plate shaker as above.

Patient group quality control (QC) samples were prepared for both serum and urine sample sets, similar to the approach described previously by Norman et al. [22]. For serum, 20 µL of each patient sample was aliquoted into a single pool. In total, five group QC pools were made: (i) Baseline, (ii) 24 months no treatment, (iii) 24 months 10 mg daily nitisinone treatment, (iv) 48 months no treatment, (v) 48 months 10 mg daily nitisinone treatment and (vi) overall pool—containing serum from all patients and visits; this acted as a system QC. QC samples were prepared as per patient samples. For urine samples, the same group QC pools were created as above, by pooling of 30 µL of each patient sample.

### 2.4. Quantitative Metabolite Data

Data from previous quantitative metabolite analyses on the same sample set described above were used in this study. Urine samples were measured for creatinine using the Jaffe creatinine assay on the Roche Cobas c701 module (product code: 06407137190, Roche diagnostics, Manheim, Germany) prior to this study [12]. Creatinine concentrations were multiplied by volume of urine collected over 24 h to give 24 h urine creatinine values; used for subsequent urine metabolite normalisation as described below.

Serum samples were measured for phenylalanine (sPHE), tyrosine (sTYR) and nitisinone (sNIT) using a published LC-MS method [26]. sPHE, sTYR and sNIT concentrations were used for correlation against metabolites measured in the present analysis.

### 2.5. LC-QTOF-MS Analysis

Analysis of serum and urine samples was performed using a published LC-QTOF-MS acquisition method [22], which employed a 1290 Infinity II HPLC coupled to a 6550 QTOF-MS equipped with dual AJS electrospray ionisation source (Agilent, Cheadle, UK). Data acquisition parameters are detailed in brief below and in full in Supplementary Methods. Reversed-phase LC was performed on an Atlantis dC18 column (3 × 100 mm, 3 μm, Waters, Manchester, UK) maintained at 60 °C. Mobile phase composition was (A) water and (B) methanol, both with 5 mmol/L ammonium formate and 0.1% formic acid. The elution gradient began at 5% B 0–1 min and increased linearly to 100% B by 12 min, held at 100% B until 14 min, then at 5% B for a further 5 min. MS data acquisition was performed in positive and negative ionisation polarity with mass range 50–1700 in 2 GHz mode with acquisition rate at 3 spectra/second. Sample injection volume was 1 and 2 µL in positive and negative polarity ionisation, respectively. The autosampler compartment was maintained at 4 °C. Data were acquired using Masshunter Acquisition (Build 06.00, Agilent, Cheadle, UK).

The analytical sequence of samples was performed according to published guidance [27]. Each run commenced with 20 replicate injections of the overall pooled sample to condition the system. The order of individual samples was randomised computationally. Pooled samples were interspersed throughout the analytical sequence every tenth injection. The analysis of serum and urine samples was divided into two separate batches; the order of individual patient samples was randomised computationally across the two batches and replicate aliquots of the same QC samples were interspersed throughout each batch sequence.

Confirmation of metabolite identifications was performed by data-dependent tandem mass spectrometry (MS2) analysis of pooled samples (pool in which the compound was most abundant), with [M + H]^+^ and [M − H]^−^ accurate mass precursor ion targets; no more than six compound targets per injection. Multiple or single fixed collision energies were applied (10–40 eV). Acquisition rates were 6 spectra/second in MS1 and 4 spectra/second in MS2.

### 2.6. Data Pre-Processing and Statistical Analysis

Raw data were mined using the targeted feature extraction function in Masshunter Profinder (build 10.00, Agilent, Cheadle, UK) with mass targets based on chemical formulae of known/predicted phe-tyr pathway metabolites from the customised compound databases described below. A combined compound database was compiled using PCDL Manager (build 08.00, Agilent, Cheadle, UK). Accurate mass retention time (AMRT) matched metabolites were present in our published AMRT database, which was generated from chemical standards using the same LC-QTOF-MS methodology employed here [28]: phenylalanine, phenylethylamine, tyrosine, *N*-acetyl-tyrosine, tyramine, HPPA, HPLA and HGA. Other established phenylalanine metabolites added to the database for mining by accurate mass alone were hydroxyphenylacetic acid, phenylacetaldehyde, phenylacetamide, phenylacetic acid, phenylacetylglutamine, phenylethylamine, phenyllactic acid and phenylpyruvic acid. The remaining formulae were from non-established but theoretically possible phase 1 and 2 biotransformation products derived from phenylalanine (n = 74), tyrosine (n = 74), HPPA (n = 67) and HPLA (n = 67) predicted using the Biotransformation Mass Defects tool (Agilent), in addition to the HGA biotransformation products (n = 7) previously established by our group [23].

Feature extraction parameters were accurate mass match window ±5 ppm with addition of matched retention time (RT; window ±0.3 min) for AMRT database metabolites. Allowed ion species were: H^+^, Na^+^, and NH_4_^+^ in positive polarity, and H^−^ and CHO_2_^−^ in negative polarity. Charge state range was 1–2, and dimers were allowed. ‘Find by formula’ filters were: score > 60 in at least 60% of samples in at least one sample group. Where compounds were detected in both positive and negative ionisation, the polarity with the clearest signal was selected for further analysis. Extracted peak area intensity data were exported in .csv file format and imported into Mass Profiler Professional (MPP; build 15.1, Agilent, Cheadle, UK), in which all statistical analyses were performed unless stated otherwise. In MPP, all data were log_2_ transformed and pareto scaled. Urine data were normalised to 24 h creatinine values.

QC was performed based on compound signal intensity data from the pooled samples interspersed throughout each analytical sequence. Compounds were retained for subsequent statistical analyses if (a) observed in 100% of replicate injections for at least one sample group pool, and (b) peak area coefficient of variation (CV) remained <30% across replicate injections for each sample group pool across batches 1 and 2 combined.

Statistical comparisons of data across sampling time points for nitisinone-treated and untreated patients were made using repeated-measures one-way ANOVA. Comparison of urine metabolite data between the sTYR threshold groups in treated patients was performed by Kruskal–Wallis test. Analysis of sex differences in urine and serum was performed by Mann–Whitney U test. Multiple testing correction of statistical significance tests was applied via Benjamini–Hochberg false discovery rate (FDR) adjustment. Hierarchical clustering analysis within patient sample groups was performed using Pearson centred similarity measure with Ward’s linkage rule.

Pearson correlation analysis was performed in Metaboanalyst version 5.0 [29] to assess relationships between urine metabolites (transformed, scaled and normalised data from MPP) and quantitative data from individual serum metabolites (sPHE, sTYR and sNIT raw concentrations) in nitisinone-treated patients.

MS2 data were processed and analysed with Masshunter Qualitative software (build 07.00, Agilent). Acquired fragmentation spectra were matched against spectra from the METLIN metabolite PCDL accurate mass library (build 07.00) where available, otherwise against in silico predicted spectra using Molecular Structure Correlator (MSC: version B.07.00, build 31) as previously described for HGA biotransformation products [23]. Spectral match thresholds (MSC scores) calculated based on multiple collision energies combined were >70% and >50% against METLIN library spectra and in silico predicted spectra (‘MSC score’), respectively.

## 3. Results

### 3.1. Summary of Metabolites Identified and Retained Post-QC Filtering

**Figure 1:**
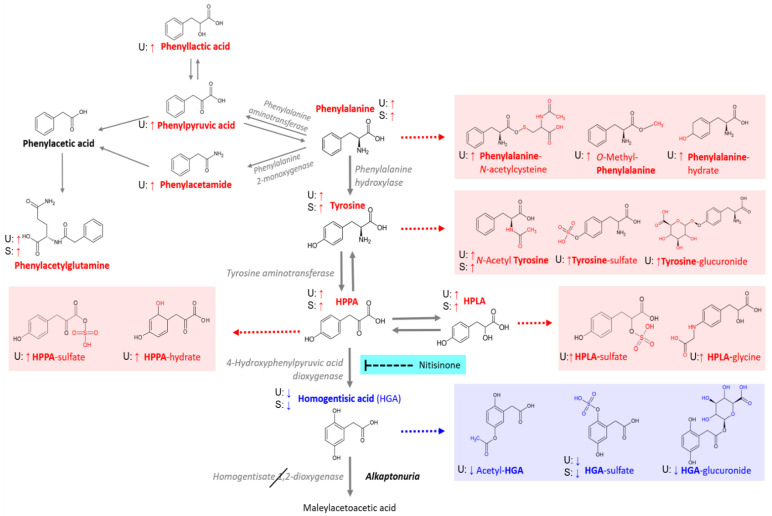
Pathway summary of metabolites identified and abundance changes in nitisinone-treated patients with AKU. Metabolites in red text, all upstream of HGA, showed increased abundance from baseline at visits 4 and 6 (2 years and 4 years on nitisinone, respectively). Metabolites associated with HGA, in blue text, decreased on nitisinone treatment. ‘U’ and ‘S’ indicates detection in urine and serum, respectively, and arrows show direction of change in each biofluid. Metabolites in red and blue boxes are biotransformation products of their respective precursor metabolites; the structures proposed for these compounds are for which the closest matches were obtained between acquired MS2 spectra and library or in silico predicted spectra.

A total of 8 metabolites in serum and 22 in urine passed QC filtering based on abundance and variability in group pool samples, in addition to presence of MS2 spectra and/or RT match supporting compound structure identifications (Table 2). In addition, urine tyramine (AMRT-matched) was included in the analysis despite not meeting the QC abundance variability filtering threshold (CV < 30%), as a clear increase in this metabolite in nitisinone-treated patients and mice with AKU has been reported in multiple studies by our group [17,22]. The metabolites that passed filtering based on QC samples as described include those of the traditional phe-tyr degradation pathway in serum and urine (phenylalanine, tyrosine, HPPA, HPLA and HGA), in addition to metabolites derived from phenylalanine by conversion to phenylpyruvic acid and phenylacetamide (Figure 1). The latter group of phenylalanine metabolites were only observed in urine, with the exception of phenylacetylglutamine, which was also observed in serum. Interestingly, among the metabolites were a number of predicted, but to our knowledge previously undescribed, phase 1 and 2 biotransformation products derived from phenylalanine, HPPA and HPLA upstream of HGA. The phase 1 products include hydrated products of phenylalanine and HPPA, observed in urine. The phase 2 products, more frequently observed in urine, include *N*-acetylcysteine and *O*-methyl conjugates of phenylalanine, glucuronide and *N*-acetyl conjugates of tyrosine, *O*-sulfate conjugates of HPPA and HPLA and a glycine conjugate of HPLA. The putative structures proposed for biotransformation compounds (Figure 1) are based on acquired MS2 fragmentation spectra (Figures S1–S5). Phenylacetaldehyde, phenylacetic acid, phenylethylamine and hydroxyphenylacetic acid were not detected or did not meet QC-based inclusion thresholds in serum or urine.

### 3.2. Effect of Nitisinone Treatment on Serum and Urine Metabolites

**Figure 2:**
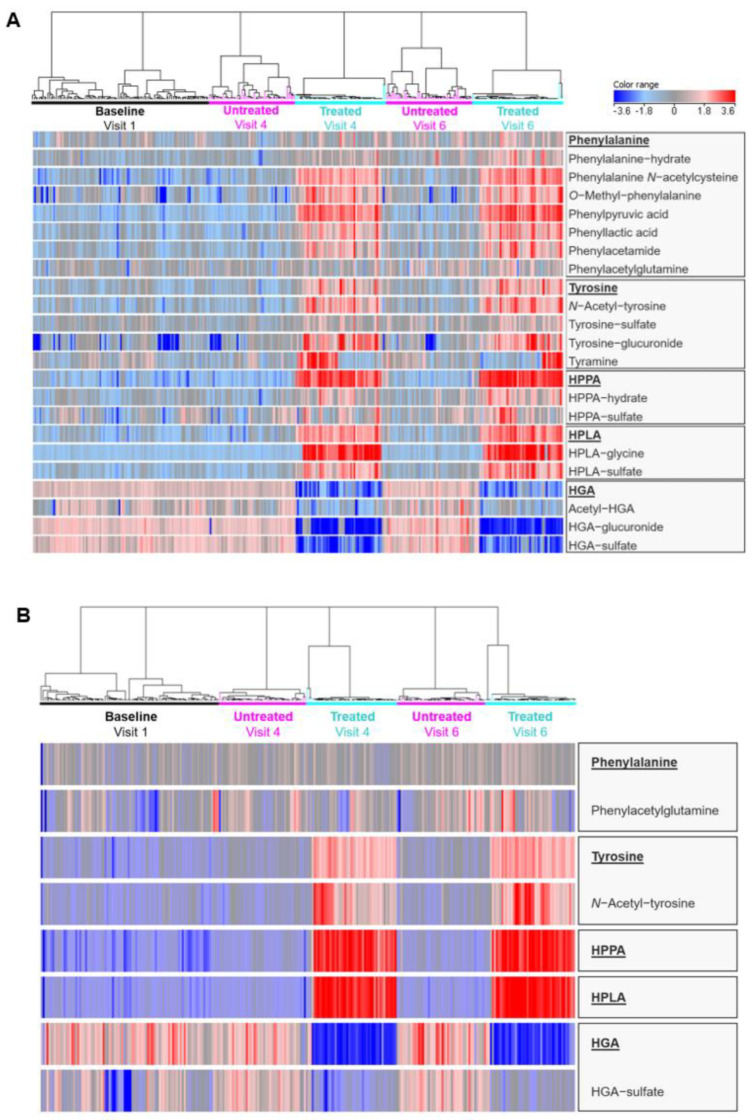
Heatmaps of urine (**A**) and serum (**B**) metabolite abundances across patient groups and sampling time points. Columns and rows represent individual samples and metabolites, respectively. Red and blue shading indicates high and low abundance, respectively. Hierarchical clustering analysis shows the sub-clustering of patients within time points for nitisinone-treated and untreated patients based on combined metabolite abundance. Figures produced using Mass Profiler Professional (build 15.1).

A marked effect of nitisinone treatment on metabolite profiles was observed in serum and urine. The heatmaps produced from hierarchical clustering analysis show clear differences between the samples taken whilst on nitisinone (visits 4 and 6 in the treated arm) compared with the baseline samples from these patients and visits 1, 4 and 6 from the untreated arm (Figure 2). Heatmap peak area intensity values show clear decreases in HGA and its associated metabolites at nitisinone-treated time points, while clear increases were observed for HPLA, HPPA and tyrosine and associated metabolites. Marked increases were also observed in urine for the phenylalanine derived metabolites, including those related to PKU and three phenylalanine biotransformation products: phenylalanine-hydrate and *N*-acetylcysteine and *O*-methyl phenylalanine conjugates.

All serum and urine metabolites studied showed statistically significant differences from baseline at visits 4 or 6 in the nitisinone-treated group at the corrected significance threshold *p* < 0.05 (Table 3). In urine, the greatest fold change (FC) against baseline in the treated group was for HPPA (FC baseline-V4 and baseline-V6 = 20 and 22.7, respectively) and for HPLA and HGA, FC from baseline ranged from 8 to 10.9. Compounds derived from the parent metabolites of the phe-tyr pathway (phenylalanine, tyrosine, HPPA, HPLA and HGA) showed considerable changes, including a number of metabolites with increased FC magnitude in comparison to their respective precursor at V4 and V6, namely *N*-acetyl-tyrosine, tyrosine glucuronide, HPLA-glycine and all phenylalanine-derived metabolites except for phenylacetylglutamine. The marked urinary increases in the phenylalanine-derived metabolites is particularly noteworthy given the relatively modest increase in urine phenylalanine on nitisinone (FC baseline-V4 and baseline-V6 = 1.2 and 1.7, respectively).

Serum data generally followed the same trends as urine, with HPPA showing the greatest increase in patients on nitisinone (FC baseline-V4 and baseline-V6 = 14.3 and 15, respectively). Of the biotransformation products detected in serum, HGA-sulfate decreased with HGA and *N*-acetyl-tyrosine increased with tyrosine.

Significant differences from baseline to visit 4 and visit 6 were also observed in the untreated group for metabolites with the exception of *O*-methyl-phenylalanine in urine and HPLA in serum. Despite these differences, the magnitude of changes observed in treated patients exceeded those of the untreated patients for each pairwise comparison from baseline for all metabolites except urine tyramine at visit 6 and serum phenylalanine at visit 4. The direction of change in metabolite abundance for the untreated group was more mixed than in the treated group in urine. Additionally, serum HGA and its sulfate conjugate increased from baseline at visits 4 and 6 in the untreated group; the opposite direction to the treated group (Table 3).

The mean raw peak areas of metabolites at each visit provide an indication of relative abundance in urine (Table S1) and serum (Table S2). It is notable that some of the biotransformation products have equal or increased abundance compared with their respective precursor metabolite (phenylalanine, tyrosine, HPPA, HPLA and HGA). Urine *N*-Acetyl-tyrosine and phenylacetylglutamine, for example, had greater mean raw abundance than their parent precursors at all time points except visit 4 in untreated patients for *N*-acetyl-tyrosine. In untreated patients, there was generally little difference between the abundance ranking order of metabolites across visits. In treated patients, there was a clear change in the abundance rankings of metabolites at visits 4 and 6 on nitisinone compared with baseline.

### 3.3. Temporal Changes in Metabolites over Long-Term Nitisinone Treatment

Interesting temporal metabolite abundance changes were observed with long-term nitisinone treatment, i.e., comparing profiles over the course of two years (visit 4) versus four years (visit 6). FC analysis (Table 3) showed that all serum and urine metabolites upstream of HGA, with the exception of urine tyrosine-glucuronide, tyramine and HPLA-glycine, showed further increases from baseline at visit 6 compared with visit 4. While there was a trend for changes from baseline across metabolites at visit 6 in the untreated group, this temporal effect was more pronounced in the treated group. Longitudinal profile plots for individual urine and serum metabolites across treated and untreated groups are shown in Figures S6 and S7.

### 3.4. Effect of Nitisinone Treatment on Summed Metabolites

The sum of all metabolites included in this analysis was calculated for serum and urine based on raw and 24 h creatinine normalised data, respectively, to indicate total pathway flux in each group/time point. In line with previous analyses [25], the serum data from treated patients showed the greatest overall differences, with total metabolite abundance markedly increasing from baseline at visit 4 (FC: treated group = 3.1; untreated group = 1.2) and visit 6 (FC: treated group = 3.6; untreated group = 1.3). The same trend of increased total metabolite abundance over successive time points on nitisinone was observed in urine, but at lower magnitude (Table 4, Figure S8).

Metabolite abundances were added together and split into groups according to their precursor compound (Table 4). Clear changes were observed in the relative contributions of each compound group to total metabolite abundance on nitisinone treatment, indicating the major contributions of tyrosine- and HPLA-derived metabolites to the total serum pathway increase in treated patients. Paired one-way ANOVAs showed statistically significant differences in total urine and serum metabolites across time points for treated and untreated patients; *p* < 0.0001 treated and untreated groups for urine and for serum in the treated group, *p* = 0.0014 for serum in the untreated group.

### 3.5. Correlation of Urine Metabolites against Quantitative Biochemical Data

**Figure 3:**
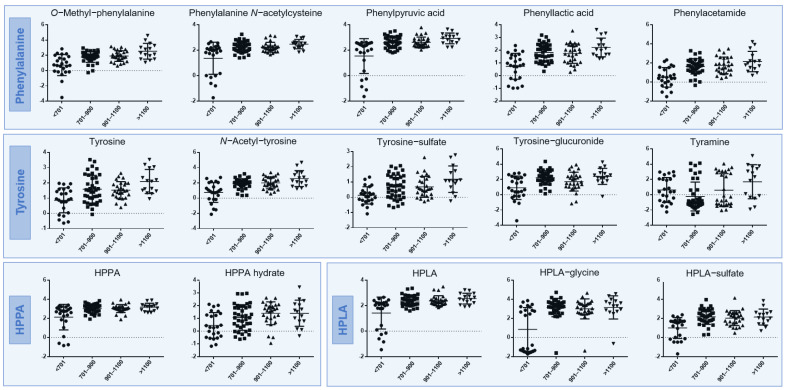
Comparison of urine metabolite abundance between corresponding sTYR concentration (µmol/L) threshold groups for samples from nitisinone-treated patients only; visits 4 and 6 combined. Data shown are from metabolites that showed statistically significant differences between sTYR groups. All data are scaled, transformed and normalised peak areas, as described. Central bars and outer bars represent mean and SD, respectively.

**Figure 4:**
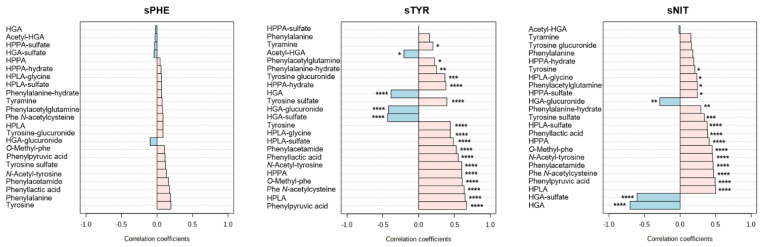
Data from Pearson correlations between urine metabolite abundance and corresponding serum biochemical concentrations of phenylalanine (sPHE), tyrosine (sTYR) and nitisinone (sNIT). Pink and blue bars represent positive and negative correlations, respectively. Data are from samples from patients on nitisinone treatment only; visits 4 and 6 combined. Figures produced using MetaboAnalyst (version 5.0) [29]. * *p* < 0.05, ** *p* < 0.01, *** *p* < 0.001, **** *p* < 0.0001.

Urine metabolite data from the time points in patients on nitisinone (visits 4 and 6 for treated patients) were compared against previous data obtained from the same samples by quantitative LC/MS [12]. The relationship between individual urine metabolites and degree of hypertyrosinaemia was investigated by comparing urine data with the corresponding serum tyrosine concentration (sTYR: µmol/L) using thresholds applied in previous analyses: <701, 701–900, 900–1100 and >1100 [31]. A total of 15 urine metabolites showed statistically significant differences between sTYR thresholds (Figure 3 and Table S3). These urine metabolites were derived from phenylalanine (N = 5), tyrosine (N = 5), HPPA (N = 2) and HPLA (N = 3). Among these metabolites there was a clear trend of increased urinary abundance with increasing sTYR concentration; a relationship confirmed by the significant (FDR-adjusted *p* < 0.05) positive correlations observed between sTYR and all urine metabolites derived from phenylalanine, tyrosine, HPPA and HPLA with the exception of phenylalanine and HPPA-sulfate. Interestingly, significant positive correlations were also observed between sNIT and all phenylalanine, tyrosine, HPPA and HPLA metabolites, with the exception of phenylalanine, tyrosine-glucuronide, tyramine and HPPA-hydrate. Conversely, significant negative correlations were observed for sTYR and sNIT against all four HGA metabolites included in this analysis, except for acetyl-HGA against sNIT. No urine metabolites showed statistically significant correlations with phenylalanine at a threshold of *p* < 0.05 (FDR-adjusted) (Figure 4).

### 3.6. Sex Differences in Metabolite Profiles

Data from patients on nitisinone (visits 4–6 combined) were compared between males and females. The only compounds with statistically significant differences were the urine metabolites phenylalanine-hydrate and phenylalanine-*N*-acetylcysteine (*p* = 0.049 for both); both decreased in males (Figure S9). Neither phenylalanine-hydrate (*p* = 0.68) nor phenylalanine-*N*-acetylcysteine (*p* = 0.53) showed a significant sex difference in the treated group at baseline. No significant sex differences were observed for the total abundances of all metabolites or summed metabolites grouped by precursor in serum or urine.

## 4. Discussion

High-resolution, high-sensitivity metabolomic approaches have previously elucidated novel biotransformation products derived from the tyrosine metabolite HGA [23]. These pathways are separate from the major phe-tyr catabolic pathway and likely represent adaptive detoxification mechanisms for HGA, the active molecule in AKU which accumulates due to inherited homogentisate 1,2-dioxygenase enzyme deficiency [32]. This study is the first comprehensive analysis of metabolite biotransformation products from phenylalanine through to HGA. The data show that alternative biotransformation pathways exist not only for HGA, but also for the metabolites upstream of HGA which are increased on nitisinone treatment; namely HPPA, HPLA, tyrosine and phenylalanine (Figure 1). In total, we observed 10 biotransformation products derived from these precursor metabolites, excluding those previously identified from HGA [23]; all 10 metabolites were observed in urine and two in serum. Of these 10 metabolites, only the *N*-acetyl conjugate of tyrosine has been reported in the disorders of phe-tyr metabolism. The increases in metabolite products were shown to be proportional to serum concentrations of tyrosine and nitisinone (Figure 3 and Figure 4) and supports the explanation that activity in these alternative metabolic pathways is increased in line with the degree of nitisinone-induced hypertyrosinaemia.

Biotransformations can be broadly divided into phase 1 and 2 metabolism. Phase 1 transformations involve the creation of new functional groups or modification of existing ones by hydrolysis, oxidation or reduction. Phase 2 transformations involve conjugation of a drug or metabolite to endogenous molecules including glucuronic acid, sulfuric acid, glutathione and amino acids [33]. The liver is the primary site of phase 1 and 2 metabolism by mass and activity, although significant activity also occurs in the kidney and intestine [34]. Phase 1 and 2 metabolism is often discussed in the context of exogenous xenobiotics including drugs and drug metabolites in pharmacology, but the role of biotransformations in inborn errors of metabolism has generally received less attention. The finding that biotransformation products exist for phenylalanine through to HGA, and that each is markedly increased in nitisinone-treated patients has important clinical implications in AKU and the other disorders affecting this part of the phe-tyr pathway including PKU, hereditary tyrosinaemia types 2 and 3 and nitisinone-treated HT-1. Addition of the newly identified metabolites to existing biochemical assays has potential to improve patient care by providing a more complete readout of pathway flux, including the clearance of toxic metabolites by alternative non-compromised pathways. The abundance values of the biotransformation products in urine (Table S1), some of which exceed those of their respective precursor metabolites, indicate a major contribution of these metabolic routes to total phe-tyr pathway flux in patients on nitisinone. The contribution of biotransformation products and the alternative metabolites of phe-tyr metabolism (excluding phenylalanine, tyrosine, HPPA, HPLA and HGA) to total measured mean abundance of all urine metabolites in treated patients is 40–41% (43–45% in untreated) compared with 59–60% (55–58% in untreated) for phenylalanine, tyrosine, HPPA, HPLA and HGA.

Hitherto no systematic investigation has surveyed the complement of theoretically possible biotransformation products in the phe-tyr pathway. Gerstman and colleagues [35] reported γ-glutamyl and *N*-acetyl conjugates of tyrosine in nitisinone-treated patients with AKU. Our new observations show that, as with HGA [23], the biotransformation products derived from phenylalanine, tyrosine, HPPA and HPLA are predominantly observed in urine (versus serum/plasma) and are dominated by phase 2 metabolic conjugation reactions with acetyl, glucuronide, glycine and sulfate groups. Sulfonation appears to be the most common biotransformation, with sulfate conjugate products observed for tyrosine, HPPA, HPLA, and HGA. The data are consistent with previous observations that xenobiotics with aromatic moieties including phenolic acids are generally rapidly detoxified to glucuronide and sulfate conjugates detectable in urine [36]. Sulfonation reactions are catalysed by enzymes belonging to the supergene family known as sulfotransferases (SULTs). SULTs can be divided into two broad groups based on location and function. Cytosolic SULTs are responsible for the metabolism of a broad range of xenobiotics and endogenous small molecule substrates. SULTs located in the Golgi apparatus are known to perform sulfonation of peptides, proteins, lipids and glycosaminoglycans, affecting structure and function of these macromolecules [37]. Free *O*-sulfate esters of tyrosine and HPPA, *p*-hydroxyphenylacetic acid and *p*-hydroxybenzaldehyde have been reported previously in urine from humans and other mammals including mouse, rabbit and rat [38,39,40]. The relative contributions of enzymatic sulfonation of L-tyrosine and turnover of tyrosine sulfated proteins to the free tyrosine-sulfate pool in urine is not known [41]. As a common post-translational modification, sulfonation of tyrosine residues in proteins has been more extensively studied than that of free L-tyrosine and is shown to occur in a wide variety of cells and tissues [42]. However, with the known marked increase in free tyrosine, in addition to HPPA and HPLA in nitisinone-treated patients, we propose that the urinary *O*-sulfate esters detected were predominantly from metabolism of the free phe-tyr metabolites as opposed to turnover of tyrosine-sulfate containing tissue macromolecules.

The findings reveal the existence of more adaptive changes in phenylalanine metabolism than previously thought in response to long-term nitisinone treatment. Phenylalanine makes a major contribution to total metabolite flux down the phe-tyr pathway; 60% of total pathway flux compared with 40% for tyrosine [43]. A previous analysis of SONIA 2 data demonstrated a gradual increase in sPHE and a decrease in the sTYR:sPHE ratio for patients on nitisinone between visits 2 (3 months) and 6 (4 years) [25]. This trend towards reduced phenylalanine-to-tyrosine conversion over time appears to indicate a progressive pathway adaptation to nitisinone-induced hypertyrosinaemia. The present data show that in addition to phenylalanine, the phenylalanine-derived metabolite phenylacetylglutamine increased in serum in treated patients across successive visits; the increase was modest but greater than the change observed in untreated patients (Table 3). The greatest temporal differences in phenylalanine metabolites were observed in urine from nitisinone-treated patients. The urinary abundance of all phenylalanine metabolites studied increased incrementally between successive visits (Table 3) and included the biotransformation metabolites phenylalanine-hydrate, phenylalanine-*N*-acetylcysteine and *O*-methyl-phenylalanine, in addition to metabolites with known urinary increases in PKU; phenylpyruvic acid, phenyllactic acid, phenylacetamide and phenylacetylglutamine [44,45,46]. In comparison, the temporal increase in urine phenylalanine measured here by LC-QTOF-MS was less marked, as previously reported from quantitative LC-MS data [25]. Together, the data indicate that increased metabolic conversion of phenylalanine and subsequent urinary elimination adds to the previously described mild PKU-like biochemical phenotype observed on long-term nitisinone treatment [25], with the likely effect of minimising phenylalanaemia.

It has been suggested that the alterations in phe-tyr metabolism mitigate adverse treatment effects in patients on nitisinone. Corneal dendritiform keratopathy is observed in a minority of nitisinone-treated patients with HT-1 and AKU [3,20,47], attributable to formation of tyrosine crystals in the cornea associated with hypertyrosinaemia. In SONIA 2, 10 out of 69 patients in the nitisinone-treated group developed keratopathy, eight of whom were male [12]. It was shown that the keratopathy group had increased tyrosine:HPLA and tyrosine:phenylalanine ratios compared with the non-keratopathy patients. These data suggest that in patients with keratopathy, impairment in the metabolic adaptations favour formation of tyrosine over other metabolites in the pathway; i.e., less inhibition of phenylalanine-to-tyrosine conversion and reduced conversion of tyrosine to HPLA. Increases in these ratios were observed in males, potentially explaining the greater incidence of keratopathy in male patients on nitisinone [48]. Other metabolism differences were observed in this study, with increases in urine phenylalanine-hydrate and phenylalanine *N*-acetylcysteine in females, suggesting that females have increased rates of phenylalanine biotransformation to these metabolites. There were no clear differences in metabolite profiles with or without keratopathy (Table S4); formal statistical analysis was not performed due to the small number of samples available for keratopathy patients (5 patients in the treated group). One potential confounder is that nitisinone dose was altered in the keratopathy patients being withdrawn temporarily for a minimum of two months and then reintroduced at a reduced dose of 2 mg daily. However, it cannot be ruled out that differences in rates of biotransformation and subsequent urinary excretion of metabolites derived from tyrosine is a determinant of keratopathy formation in nitisinone-treated patients.

The total tyrosine pathway fluxes were compared in patients with AKU and showed that the sum of serum metabolites phenylalanine, tyrosine, HPPA, HPLA and HGA (corrected for total body water) increased at four weeks on nitisinone (1, 2, 4 or 8 mg daily) in a dose-dependent manner compared with baseline measurements. In contrast, the sum of these metabolites in urine did not change from baseline over the same treatment period [49]. The increased portion of total serum metabolites on nitisinone was interpreted as the unmasking of the metabolite pool that would otherwise have implied a contribution to the formation of HGA-derived ochronotic pigment in untreated patients. As yet, it is still not possible to directly quantify total ochronotic pigment in patients as the chemical identity of ochronotic pigment is not fully understood and the pigment accumulates in tissues throughout the body in untreated AKU [50]. It is likely that these previous analyses overestimated metabolite flux towards ochronotic pigment formation by not measuring the alternative phe-tyr pathways described in the present study. However, we have now shown a similar increase in the sum of serum metabolites on nitisinone even with expanded metabolite profiles.

A limitation of our study was that the accumulated data were semi-quantitative only, due to the nature of the untargeted data acquisition. While this approach supports the inclusion of a broader range of metabolites, including those previously undescribed, the analyses of metabolite abundance based on peak area only provides approximate indications of biofluid concentration. Given the number of analytes measured, it was not practical or possible to include calibration standards for accurate quantification of each metabolite [22,51]. Due to differences in analyte chemical properties, for example ionisation efficiencies, in addition to other analytical parameters affecting signal intensity, direct comparisons of abundance values between different metabolites are not necessarily accurate. The availability of reference materials for the biotransformation products will be crucial to establish biofluid concentrations and the incorporation of these metabolites into clinical assays for a more comprehensive quantitative assessment of metabolic alteration in phe-tyr pathways. This approach will potentially help to improve the care of patients by identifying those at higher risk of complications including those associated with hypertyrosinaemia in nitisinone-treated AKU and the other metabolic disease HT-1. It is not possible to rule out the existence of the theoretically predicted biotransformation products not observed in this analysis, as it is conceivable that different analytical parameters and technologies will reveal further metabolites. In addition, it is possible that differences in protein intake between treated and untreated patients influenced phe-tyr metabolite profiles, as patients on nitisinone were advised to follow a low protein diet. Despite this likely decreased protein intake in treated patients, the phe-tyr metabolites upstream of HGA increased on nitisinone; the converse might be expected if protein intake was the main determinant of the metabolite profile changes observed.

## 5. Conclusions

In conclusion, we have shown for the first time, that phase 1 and 2 biotransformation products exist for all major metabolites in the traditional phe-tyr pathway from phenylalanine through to HGA. Each of the metabolite products upstream of HGA derived from phenylalanine, tyrosine, HPPA and HPLA showed clear increases in serum and urine in patients with AKU on nitisinone treatment. The data shed new light on the recently described temporal metabolic adaptations observed in long-term nitisinone treatment, demonstrating that such adaptations extend to products derived from the traditional phe-tyr pathway. We propose that these additional metabolite pathways, some of which have not been previously described, represent alternative routes of elimination of phe-tyr metabolites which has implications for the monitoring and management of the inherited disorders of phenylalanine and tyrosine metabolism.

## Figures and Tables

**Table 1:** Patient demographics and summary of patient numbers per trial arm and study site.

	Urine		Serum	
	Untreated	Treated	Untreated	Treated
Mean age (SD)	46.7 (9.8)	47.8 (11.2)	47.0 (9.9)	46.7 (11.3)
Total male	28	37	25	34
Total female	22	16	20	13
Total Asian	2	1	1	1
Total black	0	1	0	0
Total Caucasian	48	51	45	45
*Numbers of patients by site*				
Paris	12	15	8	11
Liverpool	14	14	14	13
Piešťany	24	24	23	23

**Table 2:** Summary of metabolite identifications. All metabolite identifications were made to identification Level 1 according to Metabolomics Standards Initiative guidelines [30], i.e., matches for at least two of the following independent orthogonal chemical properties: accurate mass (AM), tandem mass spectral (MS2) fragmentation pattern and retention time (RT).

				Urine		Serum	
Metabolite	Formula	Theoretical Neutral Monoisotopic Mass	Retention Time (RT)	Preferred Polarity	Basis for ID	Preferred Polarity	Basis for ID
Phenylalanine	C_9_H_11_NO_2_	165.0795	3.7	(+)	AM, MS2, RT	(+)	AM, MS2, RT
Phenylalanine hydrate	C_9_H_13_NO_3_	183.0869	2.2	(+)	AM, MS2 **		
Phenylalanine *N*-acetylcysteine	C_14_H_18_N_2_O_5_ S	326.0966	4.7	(−)	AM, MS2 **		
*O*-Methyl-phenylalanine	C_10_H_13_NO_2_	179.0942	4.9	(−)	AM, MS2 **		
Phenylpyruvic acid	C_9_H_8_O_3_	164.0472	4.7	(+)	AM, MS2 **		
Phenyllactic acid	C_9_H_10_O_3_	166.063	6.7	(−)	AM, MS2 **		
Phenylacetamide	C_8_H_9_NO	135.0685	4.8	(+)	AM, MS2		
Phenylacetylglutamine	C_13_H_16_N_2_O_4_	264.1114	5.5	(+)	AM, MS2 **	(+)	AM, MS2 **
Tyrosine	C_9_H_11_NO_3_	181.0756	2.2	(−)	AM, MS2, RT	(+)	AM, MS2, RT
*N*-Acetyl-tyrosine	C_11_H_13_NO_4_	223.085	4.9	(−)	AM, MS2, RT	(+)	AM, MS2, RT
Tyrosine-sulfate	C_9_H_11_NO_6_ S	261.0306	2.0	(−)	AM, MS2 **		
Tyrosine-glucuronide	C_15_H_19_NO_9_	357.1009	3.9	(+)	AM, MS2 **		
Tyramine *	C_8_H_11_NO	137.0841	2.4	(+)	AM, MS2, RT		
HPPA	C_9_H_8_O_4_	180.0425	3.7	(−)	AM, MS2, RT	(−)	AM, RT
HPPA-hydrate	C_9_H_10_O_5_	198.053	3.6	(+)	AM, MS2 **		
HPPA-sulfate	C_9_H_8_O_7_S	259.9991	5.3	(−)	AM, MS2 **		
HPLA	C_9_H_10_O_4_	182.058	4.7	(−)	AM, MS2, RT	(−)	AM, MS2, RT
HPLA-glycine	C_11_H_13_NO_5_	239.0774	4.7	(−)	AM, MS2		
HPLA-sulfate	C_9_H_10_O7_S_	262.0146	3.8	(−)	AM, MS2 **		
HGA	C_8_H_8_O_4_	168.0425	3.5	(−)	AM, MS2, RT	(−)	AM, MS2, RT
Acetyl-HGA	C_10_H_10_O_5_	210.0525	6.4	(−)	AM, MS2 **		
HGA-glucuronide	C_14_H_16_ O_10_	344.0737	2.5	(+)	AM, MS2 **		
HGA-sulfate	C_8_H_8_O_7_S	247.9991	2.8	(−)	AM, MS2 **	(+)	AM, MS2 **

* Fail QC (CV > 30% in replicate injections of at least 1 QC pooled group sample). ** MS2 match against in silico predicted spectra (otherwise against experimental library spectra).

**Table 3:** Summary of changes in urine and serum metabolite abundance between treatment groups and visits. Visits 1 (V1), 4 (V4) and 6 (V6) refer to baseline, 24 and 48 months, respectively. Patients in the treated group were on nitisinone at V4 and V6. Red arrows indicate increased abundance and blue arrows indicate decreased abundance. The *p*-value significance threshold was *p* < 0.05 (Benjamini–Hochberg false discovery rate adjusted). ‘NS’: statistically non-significant.

	Urine						Serum					
	Treated			Untreated			Treated			Untreated		
Metabolite	*p*-value (adjusted)	V4 vs. V1	V6 vs. V1	*p*-value (adjusted)	V4 vs. V1	V6 vs. V1	*p*-value (adjusted)	V4 vs. V1	V6 vs. V1	*p*-value (adjusted)	V4 vs. V1	V6 vs. V1
Phenylalanine	<0.0001	1.2 **↑**	1.7 **↑**	<0.0001	1.0 **↓**	1.4 **↑**	<0.001	1.0 **↑**	1.3 **↑**	0.0094	1.2 **↑**	1.3 **↑**
Phenylalanine hydrate	<0.0001	2.2 **↑**	3.2 **↑**	<0.001	1.0 **↓**	1.3 **↑**						
Phenylalanine- *N*-acetylcysteine	<0.0001	9.3 **↑**	10.6 **↑**	<0.0001	1.4 **↑**	1.8 **↑**						
*O*-Methyl-phenylalanine	<0.0001	5.5 **↑**	6.4 **↑**	0.062 (NS)	1.1 **↓**	1.2 **↑**						
Phenylpyruvic acid	<0.0001	11.4 **↑**	12.6 **↑**	<0.001	1.0 **↑**	1.2 **↑**						
Phenyllactic acid	<0.0001	4.9 **↑**	6.0 **↑**	0.0063	1.1 **↑**	1.3 **↑**						
Phenylacetamide	<0.0001	4.1 **↑**	5.2 **↑**	<0.0001	1.1 **↓**	1.3 **↑**						
Phenylacetylglutamine	<0.0001	1.2 **↑**	1.6 **↑**	<0.0001	1.0 **↑**	1.5 **↑**	<0.0001	1.3 **↑**	1.7 **↑**	0.0094	1.3 **↑**	1.4 **↑**
Tyrosine	<0.0001	4.0 **↑**	4.7 **↑**	<0.001	1.1 **↓**	1.2 **↑**	<0.0001	5.4 **↑**	5.7 **↑**	<0.0001	1.4 **↑**	1.4 **↑**
*N*-Acetyl-tyrosine	<0.0001	5.7 **↑**	6.8 **↑**	<0.0001	1.1 **↓**	1.2 **↑**	<0.0001	4.3 **↑**	5.4 **↑**	0.0022	1.1 **↑**	1.2 **↑**
Tyrosine-sulfate	<0.0001	1.6 **↑**	1.9 **↑**	<0.0001	1.0 **↓**	1.4 **↑**						
Tyrosine-glucuronide	<0.0001	8.0 **↑**	7.9 **↑**	0.018	1.3 **↑**	1.4 **↑**						
Tyramine	0.0028	2.1 **↑**	1.2 **↑**	0.018	1.1 **↓**	1.3 **↑**						
HPPA	<0.0001	20.0 **↑**	22.7 **↑**	<0.001	1.1 **↓**	1.1 **↑**	<0.0001	14.3 **↑**	15.0 **↑**	<0.0001	1.4 **↑**	1.5 **↑**
HPPA-hydrate	<0.0001	2.4 **↑**	3.6 **↑**	<0.0001	1.0 **↓**	1.5 **↑**						
HPPA-sulfate	<0.0001	1.7 **↑**	2.6 **↑**	<0.001	1.3 **↑**	1.6 **↑**						
HPLA	<0.0001	9.4 **↑**	10.6 **↑**	0.0063	1.0 **↑**	1.3 **↑**	<0.0001	12.8 **↑**	13.3 **↑**	0.19 (NS)	1.1 **↑**	1.1 **↑**
HPLA-glycine	<0.0001	16.2 **↑**	15.2 **↑**	<0.0001	1.0 **↓**	1.2 **↑**						
HPLA-sulfate	<0.0001	6.4 **↑**	7.9 **↑**	<0.0001	1.1 **↑**	1.4 **↑**						
HGA	<0.0001	10.9 **↓**	8.0 **↓**	<0.0001	1.0 **↓**	1.2 **↑**	<0.0001	12.0 **↓**	10.2 **↓**	0.0094	1.0 **↑**	1.3 **↑**
Acetyl-HGA	<0.0001	4.0 **↓**	2.7 **↓**	0.011	1.0 **↓**	1.3 **↑**						
HGA-glucuronide	<0.0001	18.2 **↓**	20.5 **↓**	<0.001	1.1 **↓**	1.2 **↑**						
HGA-sulfate	<0.0001	18.4 **↓**	12.1 **↓**	<0.001	1.0 **↓**	1.2 **↑**	<0.001	1.9 **↓**	1.3 **↓**	<0.0001	2.7 **↑**	2.9 **↑**

**Table 4:** Mean cumulative peak area abundance of metabolites in urine (24 h creatinine normalised only) and serum (raw non-normalised) across treatment groups and visits grouped according to respective phe-tyr precursor metabolites (phenylalanine, tyrosine, HPPA, HPLA and HGA). Visits 1, 4 and 6 refer to baseline, 24 and 48 months, respectively. Patients in the treated group were on nitisinone at visits 4 and 6. For individual contributions of all metabolites see Table S1. ‘Metabs’: metabolites.

	Untreated						Treated					
	Visit 1		Visit 4		Visit 6		Visit 1		Visit 4		Visit 6	
	Abundance	Contribution (%)	Abundance	Contribution (%)	Abundance	Contribution (%)	Abundance	Contribution (%)	Abundance	Contribution (%)	Abundance	Contribution (%)
***Urine metabolites (grouped)***												
Phenylalanine (+metabs)	4.99 × 10^6^	40.9	5.21 × 10^6^	42.5	8.68 × 10^6^	42.7	5.19 × 10^6^	40.1	7.13 × 10^6^	36.5	1.01 × 10^7^	37.7
Tyrosine (+metabs)	5.85 × 10^4^	0.5	5.16 × 10^4^	0.5	8.83 × 10^4^	0.5	6.71 × 10^4^	0.5	7.59 × 10^5^	3.8	9.37 × 10^5^	3.4
HPPA (+metabs)	2.47 × 10^4^	0.2	2.86 × 10^4^	0.3	4.64 × 10^4^	0.2	2.88 × 10^4^	0.2	3.28 × 10^6^	16.8	4.49 × 10^6^	17.4
HPLA (+metabs)	1.20 × 10^5^	1.0	1.39 × 10^5^	1.2	2.42 × 10^5^	1.1	1.19 × 10^5^	1.0	7.26 × 10^6^	39.3	9.91 × 10^6^	37.3
HGA (+metabs)	6.51 × 10^6^	57.4	6.26 × 10^6^	55.7	1.02 × 10^7^	55.4	6.97 × 10^6^	58.2	4.01 × 10^5^	3.6	6.94 × 10^5^	4.3
Total urine metabolites	1.17 × 10^7^	-	1.17 × 10^7^	-	2.28 × 10^7^	-	1.24 × 10^7^	-	1.88 × 10^7^	-	2.62 × 10^7^	-
***Serum metabolites (grouped)***												
Phenylalanine (+metabs)	1.61 × 10^7^	79.9	1.83 × 10^7^	76.7	2.00 × 10^7^	76.9	1.58 × 10^7^	79.5	1.64 × 10^7^	28.5	2.06 × 10^7^	31.2
Tyrosine (+metabs)	3.25 × 10^6^	16.3	4.81 × 10^6^	20.4	5.14 × 10^6^	19.3	3.19 × 10^6^	16.3	3.53 × 10^7^	56.5	3.85 × 10^7^	53.7
HPPA (+metabs)	3.12 × 10^3^	0.0	5.72 × 10^3^	0.0	6.48 × 10^3^	0.0	2.99 × 10^3^	0.0	5.76 × 10^5^	0.9	7.00 × 10^5^	0.9
HPLA (+metabs)	5.24 × 10^4^	0.4	5.52 × 10^4^	0.3	5.83 × 10^4^	0.2	4.90 × 10^4^	0.3	9.00 × 10^6^	14.0	1.04 × 10^7^	13.9
HGA (+metabs)	6.38 × 10^5^	3.5	6.09 × 10^5^	2.6	9.98 × 10^5^	3.6	7.28 × 10^5^	3.8	3.91 × 10^4^	0.2	7.25 × 10^4^	0.3
Total serum metabolites	2.01 × 10^7^	-	2.38 × 10^7^	-	2.62 × 10^7^	-	1.98 ×10^7^	-	6.13 × 10^7^	-	7.04 × 10^7^	-

## Data Availability

The raw and extracted metabolomic data from this study have been uploaded to Metabolomics Workbench* and can be accessed directly by the project (PR001471) DOI: http://dx.doi.org/10.21228/M8KH70. Urine dataset study ID: ST002297; Serum dataset study ID: ST002296. *Metabolomics Workbench: An international repository for metabolomics data and metadata, metabolite standards, protocols, tutorials and training, and analysis tools (2016)’. [PubMed: https://www.ncbi.nlm.nih.gov/pubmed/26467476/].

## Supplementary Materials

The following supporting information can be downloaded at: https://www.mdpi.com/article/10.3390/metabo12100927/s1, Figure S1: representative fragmentation spectra acquired from biotransformation products derived from phenylalanine; Figure S2: representative fragmentation spectra acquired from biotransformation products derived from tyrosine; Figure S3: representative fragmentation spectra acquired from biotransformation products derived from 4-hydroxyphenylpyruvic acid (HPPA); Figure S4: representative fragmentation spectra acquired from biotransformation products derived from 4-hydroxyphenyllactic acid (HPLA); Figure S5: representative fragmentation spectra acquired from biotransformation products derived from homogentisic acid (HGA); Figure S6: longitudinal profile plots for urine metabolites across visits 1 (baseline), 4 and 6; Figure S7: longitudinal profile plots for serum metabolites across visits 1 (baseline), 4 and 6; Figure S8: longitudinal profile plots for total summed urine (A) and serum (B) metabolites across visits 1 (baseline), 4 and 6; Figure S9: sex differences in urine metabolites among the nitisinone-treated group; Table S1: mean raw peak area abundance of individual urine metabolites (24-hr creatinine scaled only) across visits 1,4 and 6 in treated and untreated patients; Table S2: mean raw peak area abundance of individual serum metabolites (raw non-normalised) across treatment groups and visits; Table S3: comparison of urine metabolites showing statistically significant (FDR-adjusted *p* < 0.05) differences between serum tyrosine (sTYR) threshold groups in patients on nitisinone (visits 4–6 samples combined); Table S4: comparison of urine metabolites between patients in the treated group who developed tyrosinaemia-associated corneal dendritiform keratopathy (KP; N = 5) and those who did not (N = 48); Supplementary Methods; quadrupole time-of-flight mass spectrometry (QTOF-MS) conditions.
