# Supplementary material for: Comprehensive Biotransformation Analysis of Phenylalanine-Tyrosine Metabolism Reveals Alternative Routes of Metabolite Clearance in Nitisinone-Treated Alkaptonuria
Source: Metabolites. 2022 Sep 29;12(10):927. doi: 10.3390/metabo12100927 (PMC9611790; doi:10.3390/metabo12100927)
Supplement: Supplementary file 1 [file metabolites-12-00927-s001.zip › Supplementary Figures.pdf]

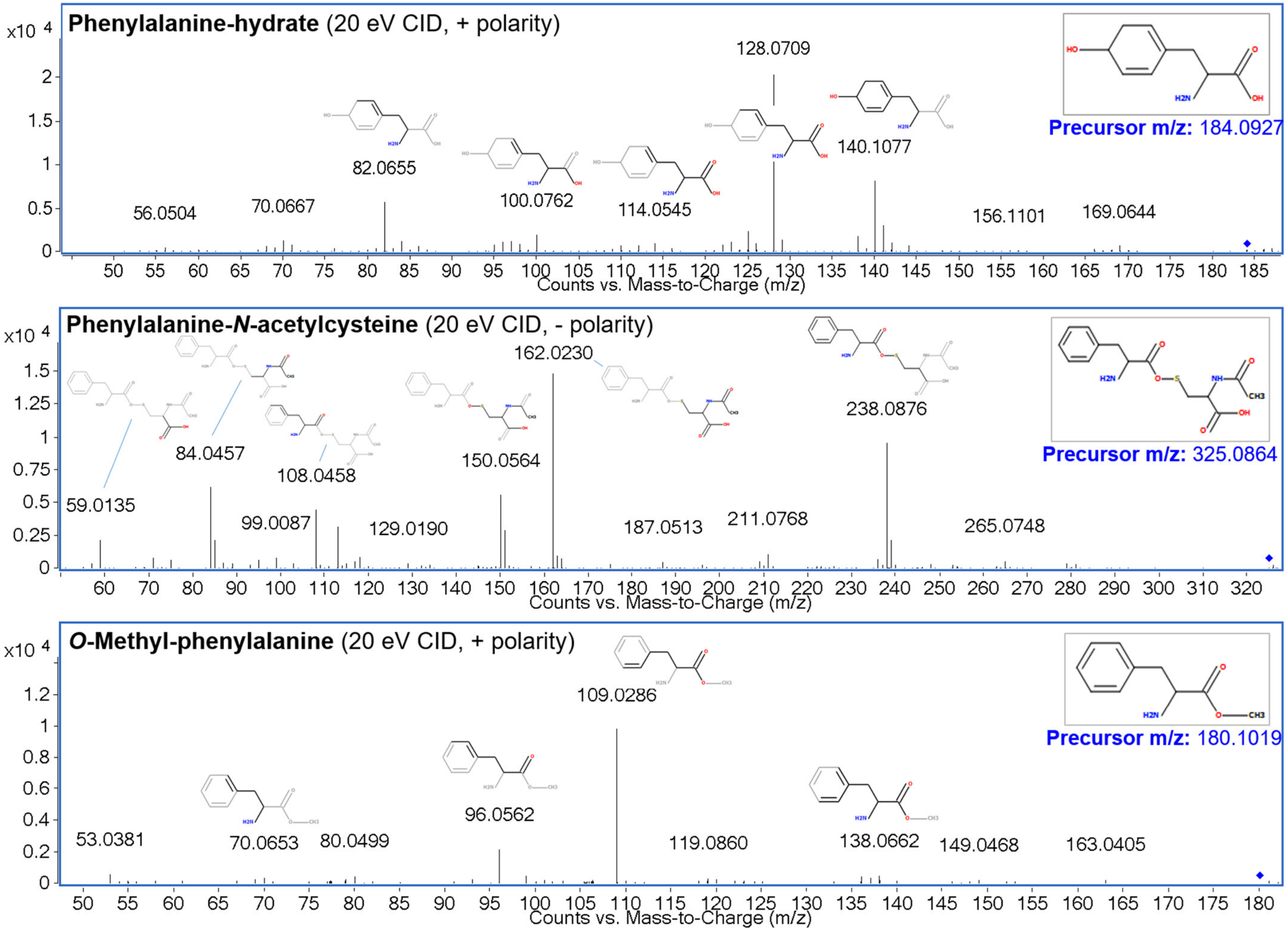

Figure S1. Representative fragmentation spectra acquired from biotransformation products derived from phenylalanine. The spectra shown were acquired from targeted MS2 analysis of pooled urine; blue diamond icons indicate the *m*/*z* of selected fragmented precursor ions. The highlighted sub-structures assigned to fragment ions were considered the most likely matches obtained from *in silico* compound fragmentation using Agilent Molecular Structure Correlator (MSC). MSC match scores were 68.8 % (phenylalanine-hydrate), 66.8 % (phenylalanine *N*-actylcysteine) and 54.6 % (O-methyl-phenylalanine). CID: collision-induced dissociation.

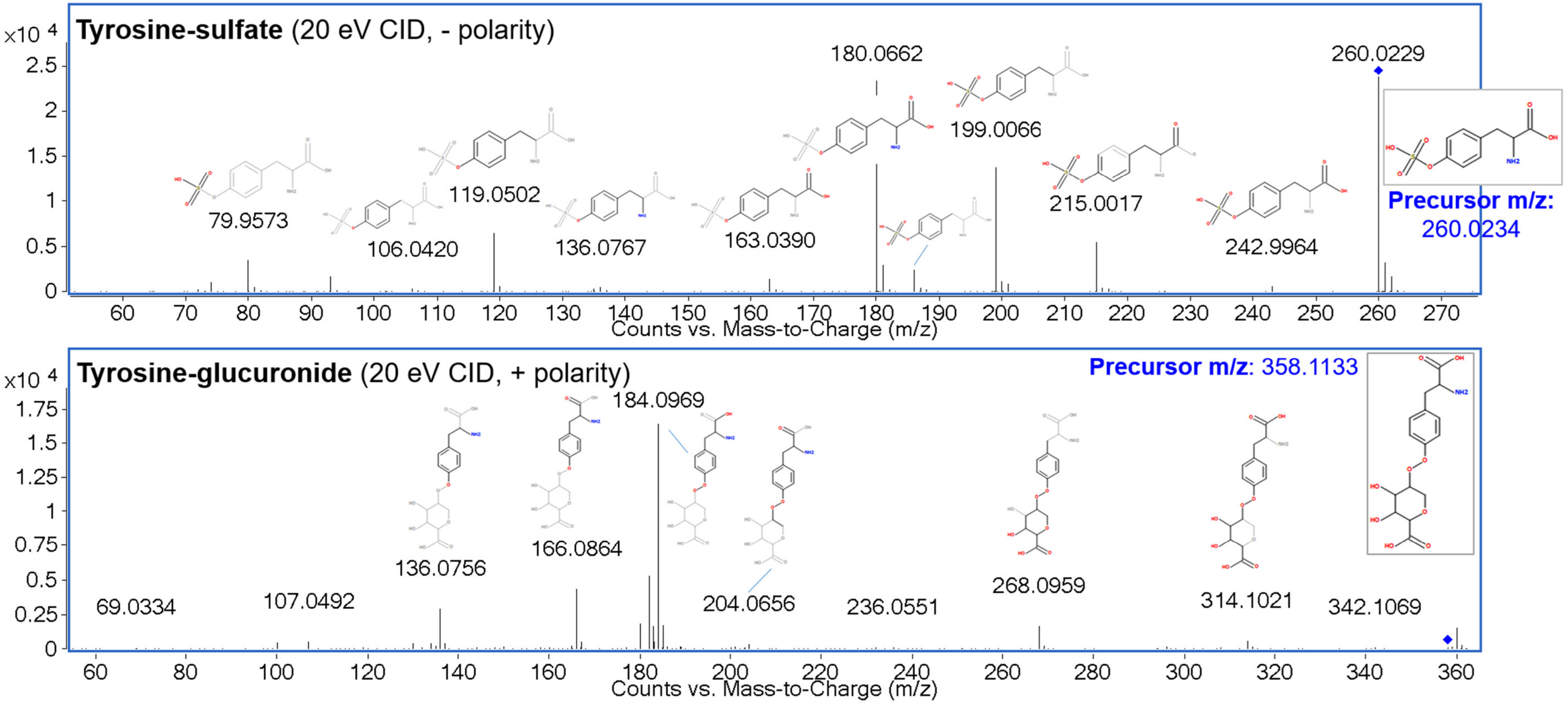

Figure S2. Representative fragmentation spectra acquired from biotransformation products derived from tyrosine. The spectra shown were acquired from targeted MS2 analysis of pooled urine; blue diamond icons indicate the *m/z* of selected fragmented precursor ions. Sub-structures assigned to fragment ions were considered the most likely matches obtained from *in silico* compound fragmentation using Agilent Molecular Structure Correlator (MSC). MSC match scores were 83.4 % (tyrosine sulfate) and 72.4 % (tyrosine-glucuronide). CID: collision-induced dissociation.

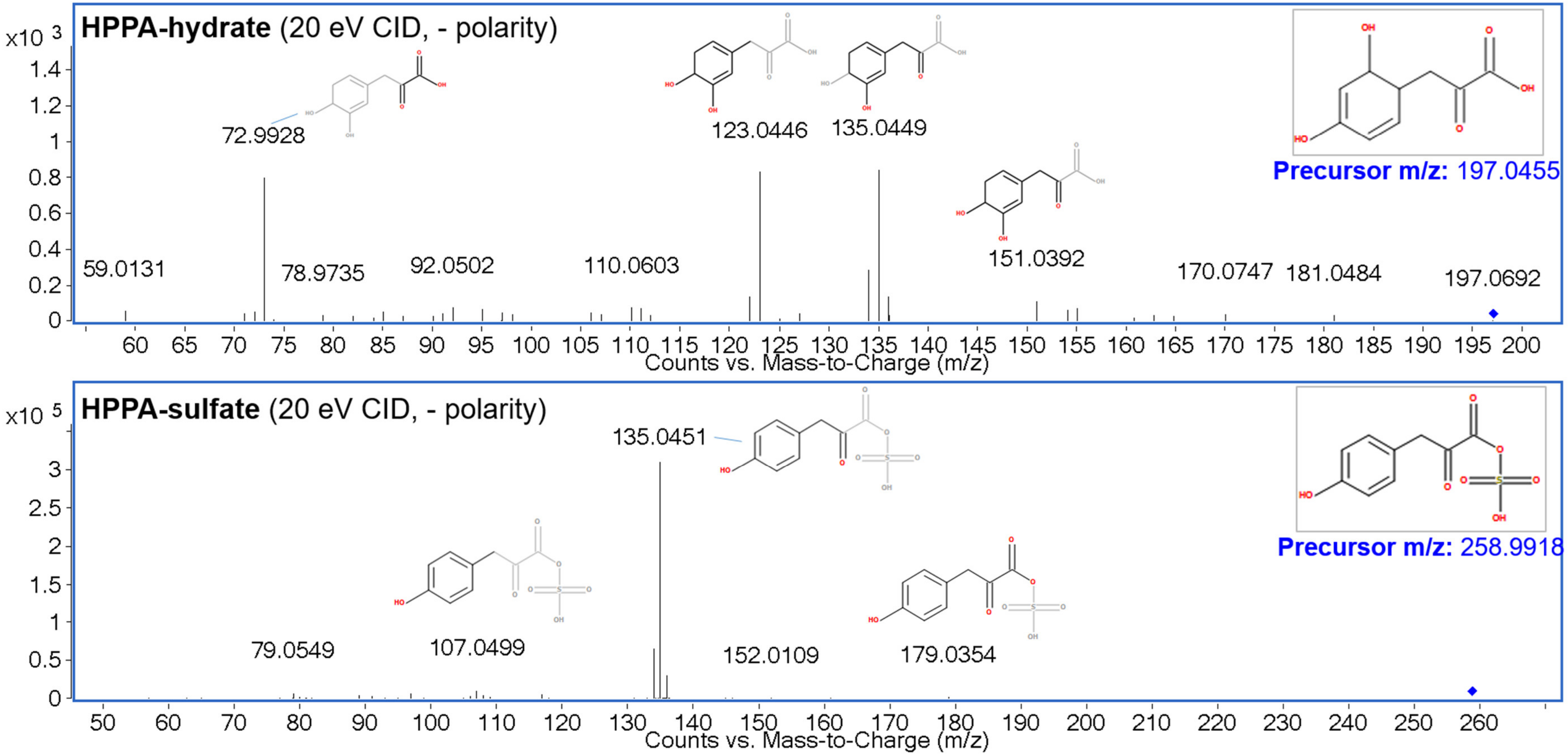

Figure S3. Representative fragmentation spectra acquired from biotransformation products derived from 4-hydroxyphenylpyruvic acid (HPPA). The spectra shown were acquired from targeted MS2 analysis of pooled urine; blue diamond icons indicate the *m/z* of selected fragmented precursor ions. Sub-structures assigned to fragment ions were considered the most likely matches obtained from *in silico* compound fragmentation using Agilent Molecular Structure Correlator (MSC). MSC match scores were 78.4 % (HPPA-hydrate) and 89.1 % (HPPA-sulfate). CID: collision-induced dissociation.

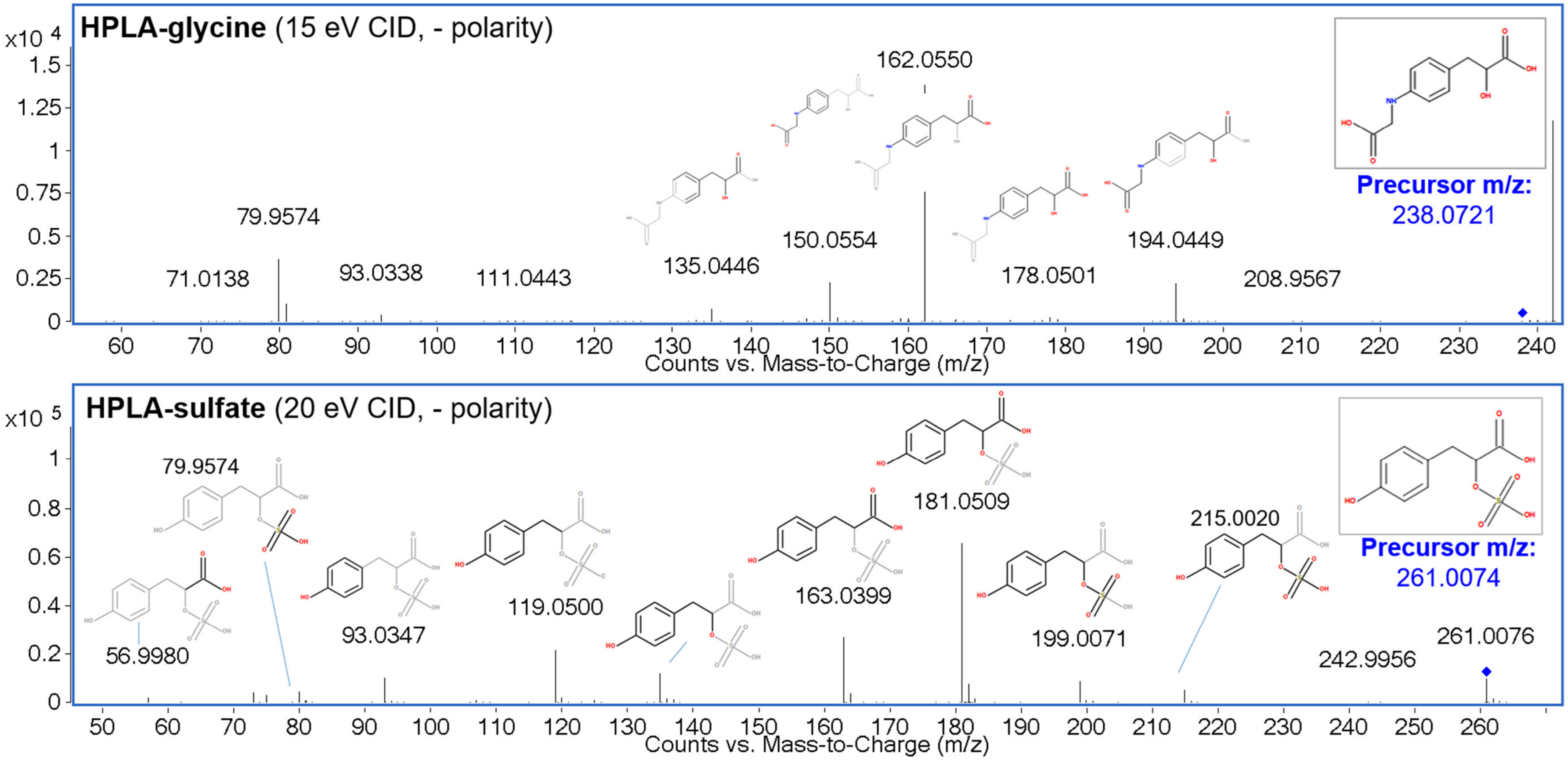

Figure S4. Representative fragmentation spectra acquired from biotransformation products derived from 4-hydroxyphenyllactic acid (HPLA). The spectra shown were acquired from targeted MS2 analysis of pooled urine; blue diamond icons indicate the *m/z* of selected fragmented precursor ions. Sub-structures assigned to fragment ions were considered the most likely matches obtained from *in silico* compound fragmentation using Agilent Molecular Structure Correlator (MSC). MSC match scores were 68.8 % (HPLA-glycine) and 89.6 % (HPLA-sulfate). CID: collision-induced dissociation.

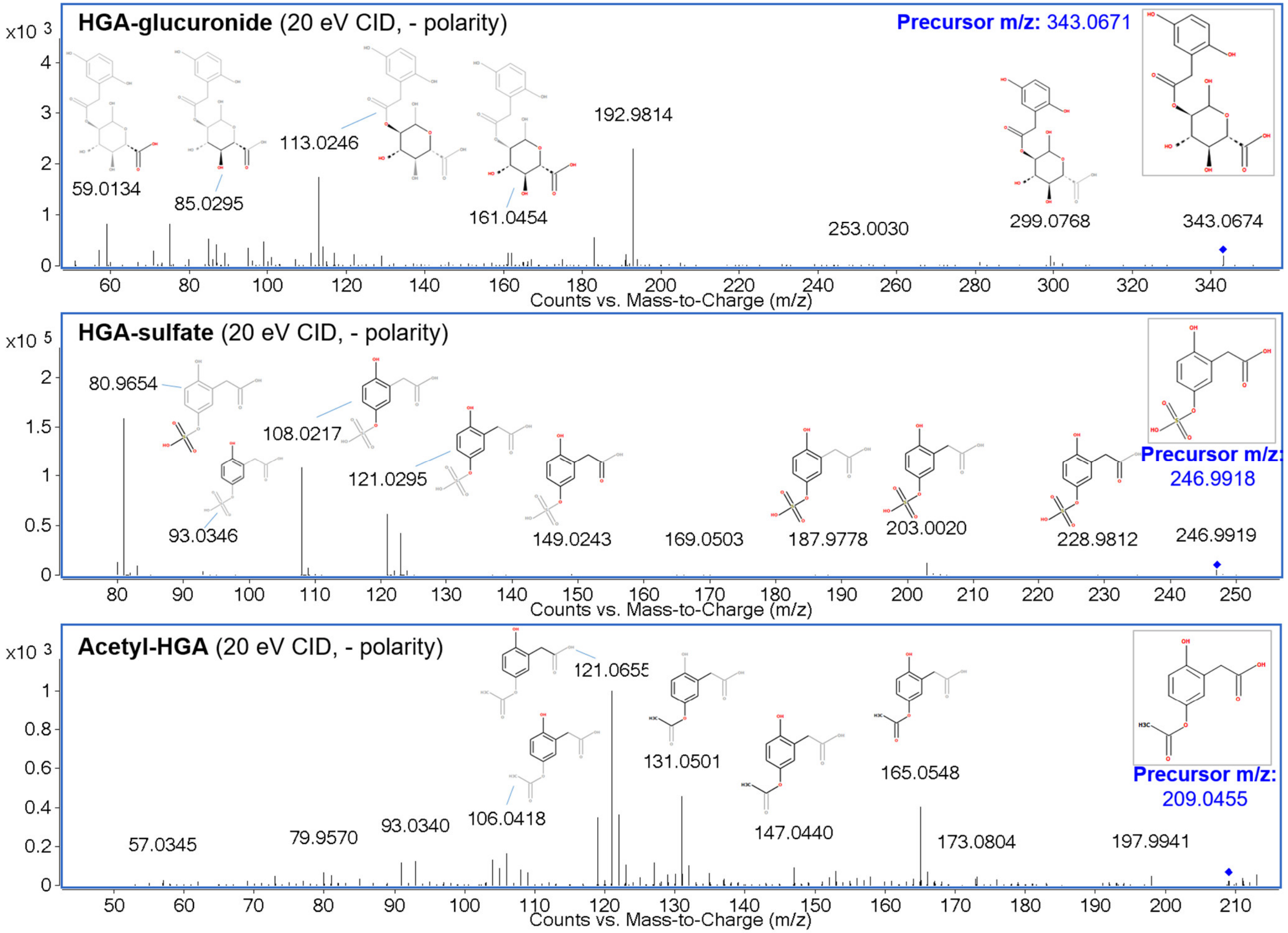

Figure S5. Representative fragmentation spectra acquired from biotransformation products derived from homogentisic acid (HGA). The spectra shown were acquired from targeted MS2 analysis of pooled urine; blue diamond icons indicate the *m*/*z* of selected fragmented precursor ions. Sub-structures assigned to fragment ions were considered the most likely matches obtained from *in silico* compound fragmentation using Agilent Molecular Structure Correlator (MSC). MSC match scores were 60.4 % (HGA-glucuronide), 85.2 % (HGA sulfate) and 60.2 % (HGA-sulfate). CID: collision-induced dissociation.

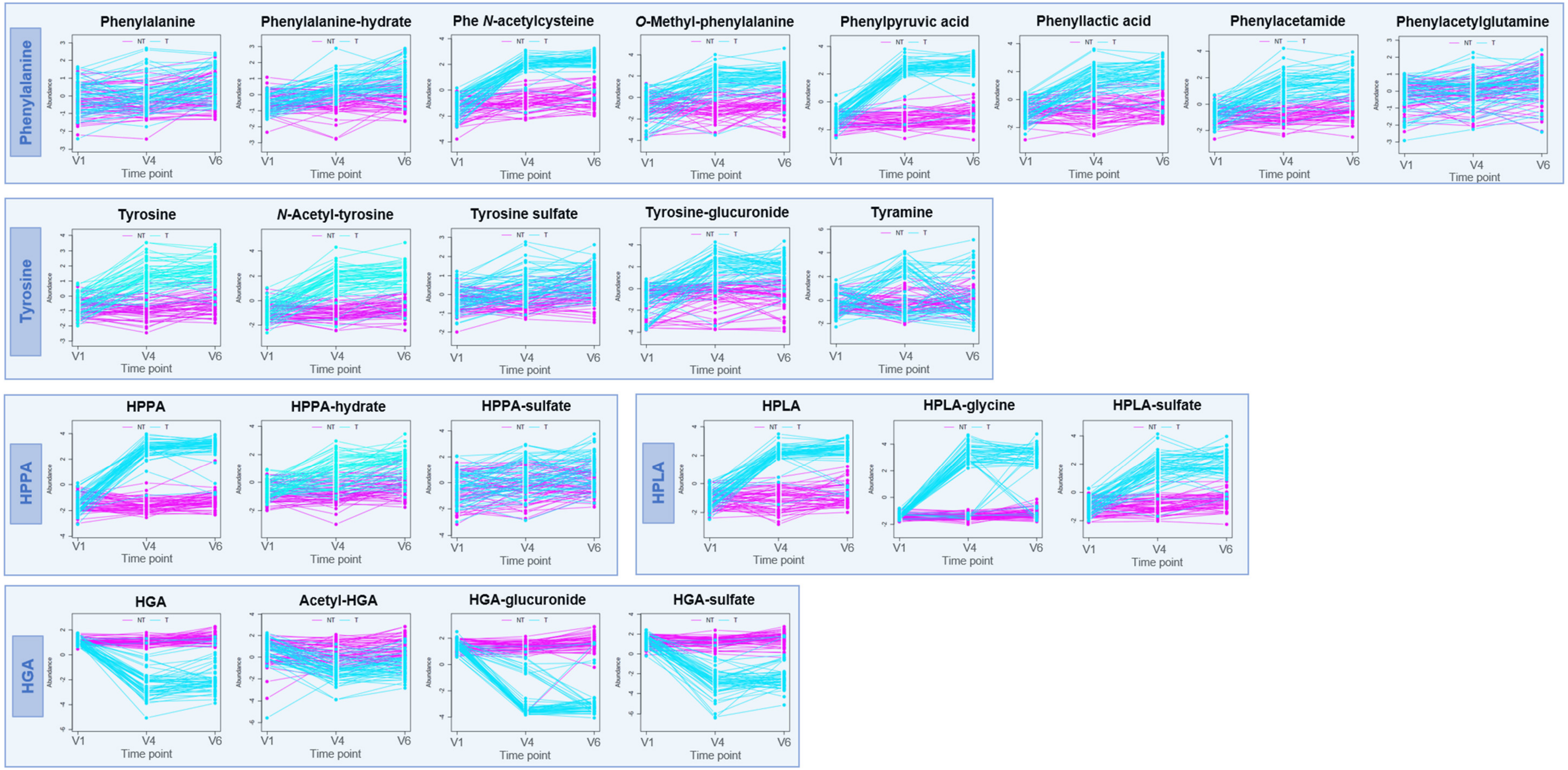

Figure S6. Longitudinal profile plots for urine metabolites across visits 1 (baseline), 4 and 6. Lines represent temporal profiles of metabolite abundance for individual patients in nitisinone-treated (blue) and untreated (pink) patients. All data are scaled, transformed and normalised peak areas, as described in Materials and Methods. Visits 1 (V1), 4 (V4) and 6 (V6) refer to baseline, 24 and 48 months respectively. Patients in the treated group were on nitisinone at V4 and V6.

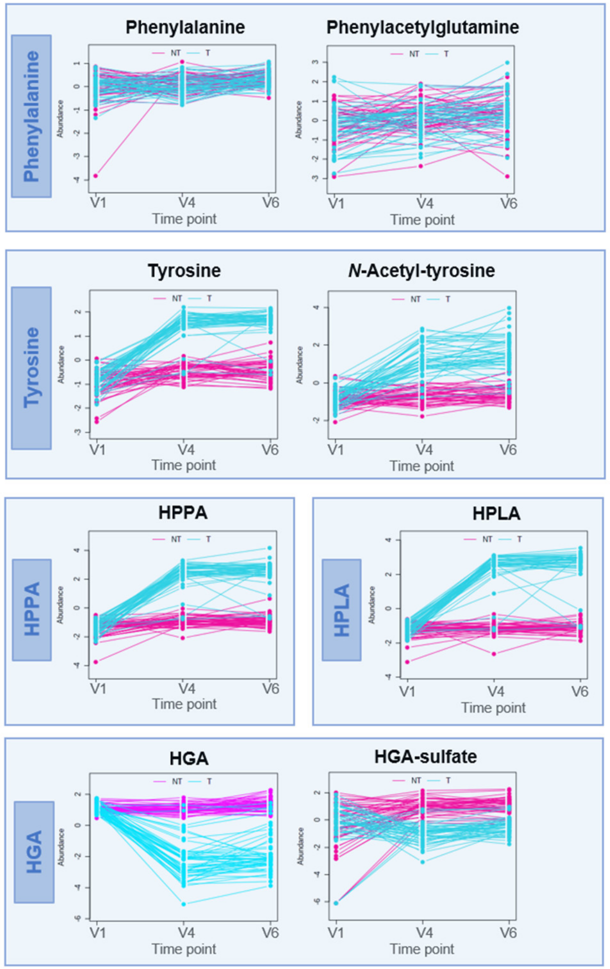

Figure S7. Longitudinal profile plots for serum metabolites across visits 1 (baseline), 4 and 6. Lines represent temporal profiles of metabolite abundance for individual patients in nitisinone-treated (blue) and untreated (pink) patients. All data are scaled, transformed and normalised peak areas, as described in Materials and Methods. Visits 1 (V1), 4 (V4) and 6 (V6) refer to baseline, 24 and 48 months respectively. Patients in the treated group were on nitisinone at V4 and V6.

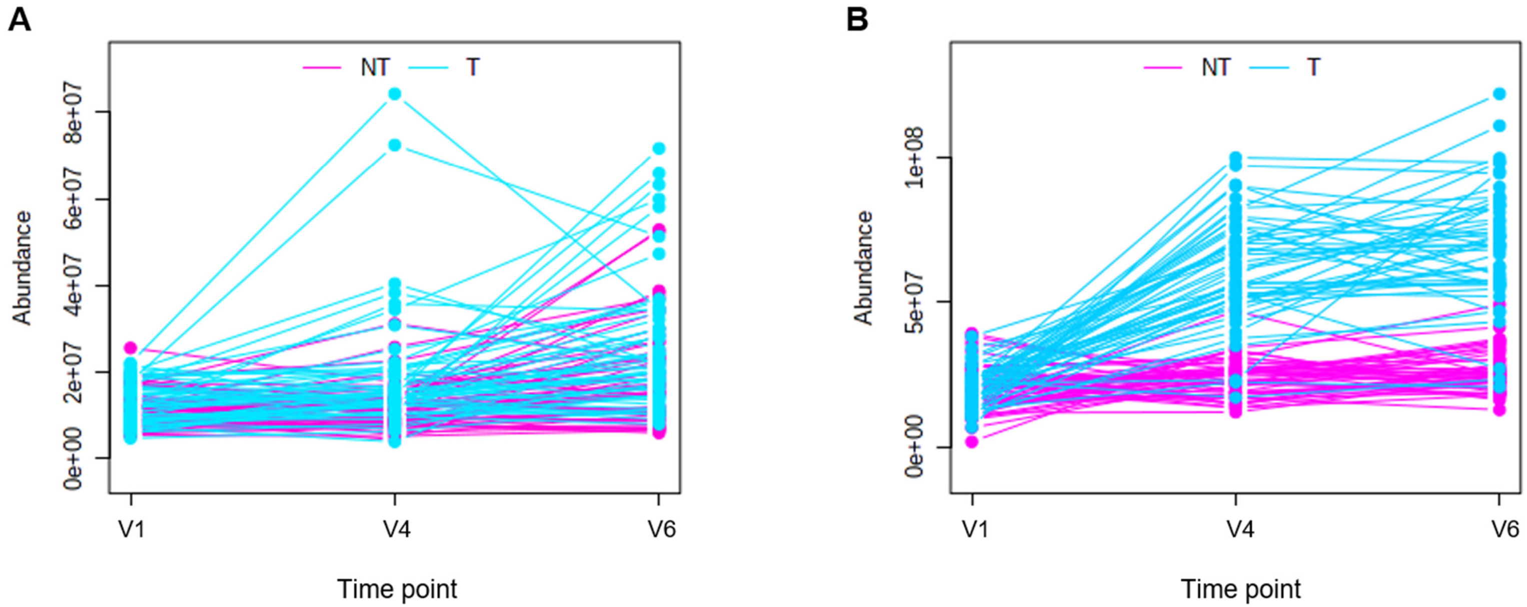

Figure S8. Longitudinal profile plots for total summed urine (A) and serum (B) metabolites across visits 1 (baseline), 4 and 6. Lines represent temporal profiles of metabolite abundance for individual patients in nitisinone-treated (blue) and untreated (pink) patients. Urine data are 24-h creatinine normalised only. Serum data are raw. Visits 1 (V1), 4 (V4) and 6 (V6) refer to baseline, 24 and 48 months respectively. Patients in the treated group were on nitisinone at V4 and V6.

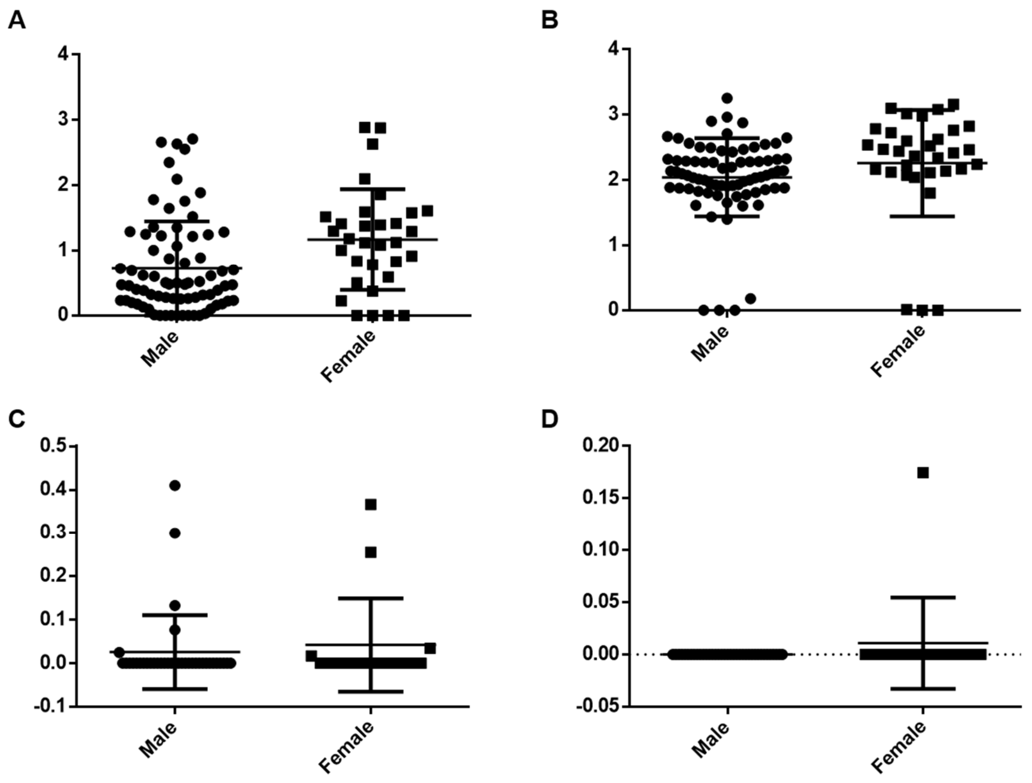

Figure S9. Sex differences in urine metabolites among the nitisinone-treated group. Dot plots of data from urine metabolites phenylalanine-hydrate and phenylalanine *N*-acetylcysteine, which showed significant differences (Benjamini-Hochberg FDR-adjusted $p < 0.05$) between males and females on nitisinone treatment at visits 4 (24 months) and 6 (48 months) combined but no significant differences in the same patients at baseline (visit 1). A – phenylalanine-hydrate on nitisinone; B - phenylalanine *N*-acetylcysteine on nitisinone; C - phenylalanine-hydrate at baseline; D - phenylalanine *N*-acetylcysteine at baseline.
