# Supplementary material for: Comprehensive Biotransformation Analysis of Phenylalanine-Tyrosine Metabolism Reveals Alternative Routes of Metabolite Clearance in Nitisinone-Treated Alkaptonuria
Source: Metabolites. 2022 Sep 29;12(10):927. doi: 10.3390/metabo12100927 (PMC9611790; doi:10.3390/metabo12100927)
Supplement: Supplementary file 1 [file metabolites-12-00927-s001.zip › Supplementary Methods.pdf]

Quadrupole time-of-flight mass spectrometry (QTOF-MS) conditions

An Agilent 6550 QTOF-MS, equipped with a dual jet stream electrospray ionisation source, was operated in 2 GHz mode, over the mass range of 50–1700, in negative and positive polarities. A reference mass correction solution was continually infused at a flow rate of 0.5 mL/min via an external isocratic pump (Agilent, Cheadle, UK) for constant mass correction (see preparation of reference mass correction solution). Capillary and fragmentor voltages were 4000 V and 380 V, respectively. Desolvation gas temperature was 200 °C with flow rate at 15 L/min. The sheath gas temperature was 300 °C with flow rate at 12 L/min, and nebulizer pressure was 40 psi and nozzle voltage 1000 V. Data acquisition rate was 3 spectra/s.

Preparation of reference mass correction solution

Reference mass correction solution was prepared in 95:5 methanol:water containing 5 mmol/L purine (CAS No. 120-73-0), 100 mmol/L trifluoroacetic acid ammonium salt (TFA, CAS No. 3336-58-1) and 2.5 mmol/L hexakis(1H, 1H, 3H-tetrafluoropropoxy)phosphazine (HP-0921, CAS No. 58943-98-9) (Agilent, Cheadle, UK).

Reference ions monitored were: purine (m/z 121.0509) and HP-0921 (m/z 922.0098) (positive polarity) and TFA (m/z 112.9856), purine (m/z 119.0363) and HP-0921 (HP-0921 + formate adduct: m/z 966.0007)(negative polarity).
