# Supplementary material for: Comprehensive Biotransformation Analysis of Phenylalanine-Tyrosine Metabolism Reveals Alternative Routes of Metabolite Clearance in Nitisinone-Treated Alkaptonuria
Source: Metabolites. 2022 Sep 29;12(10):927. doi: 10.3390/metabo12100927 (PMC9611790; doi:10.3390/metabo12100927)
Supplement: Supplementary file 1 [file metabolites-12-00927-s001.zip › Supplementary Tables.pdf]

Table S1. Mean raw peak area abundance of individual urine metabolites (24-hr creatinine scaled only) across visits 1,4 and 6 in treated and untreated patients. Visits 1, 4 and 6 refer to baseline, 24 and 48 months respectively. Patients in the treated group were on nitisinone at visits 4 and 6.

		Untreated						Treated					
		Visit 1		Visit 4		Visit 6		Visit 1		Visit 4		Visit 6	
Metabolite	Metabolite group	Mean	Rank	Mean	Rank	Mean	Rank	Mean	Rank	Mean	Rank	Mean	Rank
Phenylalanine	Phenylalanine	1.9 x10^5	3	1.8 x10^5	3	3.0 x10^5	3	2.29 x10^5	3	3.14 x10^5	6	4.23 x10^5	6
Phenylalanine hydrate	Phenylalanine	6.8 x10^3	14	6.5 x10^3	13	1.0 x10^4	15	5.94 x10^3	15	1.42 x10^4	16	2.32 x10^4	16
Phenylalanine *N*-acetylcysteine	Phenylalanine	7.8 x10^2	19	1.3 x10^3	19	2.2 x10^3	19	8.49 x10^2	20	3.74 x10^4	15	5.03 x10^4	14
O-Methyl-phenylalanine	Phenylalanine	<100	22	<100	22	<100	22	<100	22	1.23 x10^3	22	1.54 x10^3	22
Phenylpyruvic acid	Phenylalanine	7.4 x10^2	20	8.0 x10^2	20	1.3 x10^3	20	1.00 x10^3	19	1.04 x10^5	9	1.32 x10^5	9
Phenyllactic acid	Phenylalanine	1.7 x10^4	8	2.2 x10^4	7	2.9 x10^4	6	1.76 x10^4	10	2.53 x10^5	7	3.31 x10^5	7
Phenylacetamide	Phenylalanine	7.5 x10^3	12	6.5 x10^3	14	1.1 x10^4	14	8.43 x10^3	13	7.86 x10^4	11	1.01 x10^5	11
Phenylacetylglutamine	Phenylalanine	4.8 x10^6	2	5.0 x10^6	2	8.3 x10^6	2	4.92 x10^6	2	6.32 x10^6	2	9.08 x10^6	2
Tyrosine	Tyrosine	1.6 x10^4	9	1.5 x10^4	9	2.3 x10^4	10	1.93 x10^4	9	1.80 x10^5	8	2.07 x10^5	8
N-Acetyl-tyrosine	Tyrosine	1.9 x10^4	6	1.4 x10^4	10	2.7 x10^4	8	2.03 x10^4	8	4.73 x10^5	4	6.24 x10^5	5
Tyrosine sulfate	Tyrosine	1.8 x10^4	7	1.7 x10^4	8	2.9 x10^4	7	2.07 x10^4	6	4.21 x10^4	14	4.84 x10^4	15
Tyrosine glucuronide	Tyrosine	<100	21	1.3 x10^2	21	1.7 x10^2	21	<100	21	4.84 x10^3	18	4.13 x10^3	19

Tyramine	Tyrosine	5.5 x10^{3}	16	5.7 x10^{3}	15	9.7 x10^{3}	16	6.71 x10^{3}	14	5.97 x10^{4}	12	5.35 x10^{4}	13
HPPA	HPPA	6.5 x10^{3}	15	4.5 x10^{3}	16	1.8 x10^{4}	11	5.87 x10^{3}	16	3.21 x10^{6}	3	4.38 x10^{6}	3
HPPA-hydrate	HPPA	2.1 x10^{3}	18	2.1 x10^{3}	18	3.6 x10^{3}	18	2.43 x10^{3}	18	8.62 x10^{3}	17	1.31 x10^{4}	17
HPPA-sulfate	HPPA	1.6 x10^{4}	10	2.2 x10^{4}	6	2.5 x10^{4}	9	2.05 x10^{4}	7	5.55 x10^{4}	13	9.78 x10^{4}	12
HPLA	HPLA	1.2 x10^{5}	4	1.4 x10^{5}	4	2.4 x10^{5}	4	1.16 x10^{5}	5	7.18 x10^{6}	1	9.80 x10^{6}	1
HPLA-glycine	HPLA	<100	23	<100	23	<100	23	<100	23	2.04 x10^{3}	20	1.74 x10^{3}	21
HPLA sulfate	HPLA	2.5 x10^{3}	17	2.9 x10^{3}	17	4.5 x10^{3}	17	2.82 x10^{3}	17	8.44 x10^{4}	10	1.04 x10^{5}	10
HGA	HGA	6.4 x10^{6}	1	6.2 x10^{6}	1	1.0 x10^{7}	1	6.82 x10^{6}	1	3.96 x10^{5}	5	6.81 x10^{5}	4
Acetyl-HGA	HGA	7.5 x10^{3}	13	6.9 x10^{3}	12	1.2 x10^{4}	13	8.90 x10^{3}	12	1.35 x10^{3}	21	2.44 x10^{3}	20
HGA-glucuronide	HGA	8.6 x10^{3}	11	7.5 x10^{3}	11	1.5 x10^{4}	12	9.50 x10^{3}	11	3.46 x10^{2}	23	6.03 x10^{2}	23
HGA sulfate	HGA	1.0 x10^{5}	5	9.3 x10^{4}	5	1.7 x10^{5}	5	1.31 x10^{5}	4	3.27 x10^{3}	19	1.06 x10^{4}	18

Table S2. Mean raw peak area abundance of individual serum metabolites (raw non-normalised) across treatment groups and visits. Visits 1, 4 and 6 refer to baseline, 24 and 48 months respectively. Patients in the treated group were on nitisinone at visits 4 and 6.

		Untreated						Treated					
		Visit 1		Visit 4		Visit 6		Visit 1		Visit 4		Visit 6	
Metabolite	Metabolite group	Mean	Rank	Mean	Rank	Mean	Rank	Mean	Rank	Mean	Rank	Mean	Rank
Phenylalanine	Phenylalanine	1.5 x10^7	1	1.7 x10^7	1	1.9 x10^7	1	1.5 x10^7	1	1.5 x10^7	2	1.9 x10^7	2
Phenylacetylglutamine	Phenylalanine	9.1 x10^5	3	1.3 x10^6	3	1.5 x10^6	3	9.4 x10^5	3	1.1 x10^6	4	1.7 x10^6	4
Tyrosine	Tyrosine	3.2 x10^6	2	4.8 x10^6	2	5.1 x10^6	2	3.2 x10^6	2	3.5 x10^7	1	3.8 x10^7	1
N-Acetyl tyrosine	Tyrosine	1.6 x10^4	6	1.7 x10^4	6	2.0 x10^4	6	1.6 x10^4	6	1.2 x10^5	6	1.7 x10^5	6
HPPA	HPPA	3.1 x10^3	7	5.7 x10^3	8	6.5 x10^3	7	3.0 x10^3	7	5.8 x10^5	5	7.0 x10^5	5
HPLA	HPLA	5.2 x10^4	5	5.5 x10^4	5	5.8 x10^4	5	4.9 x10^4	5	9.0 x10^6	3	1.0 x10^7	3
HGA	HGA	6.3 x10^5	4	6.0 x10^5	4	9.9 x10^5	4	7.3 x10^5	4	3.8 x10^4	7	7.1 x10^4	7
HGA-sulfate	HGA	3.1 x10^3	8	5.9 x10^3	7	6.5 x10^3	8	3.0 x10^3	8	1.0 x10^3	8	1.6 x10^3	8

Table S3. Comparison of urine metabolites showing statistically significant (FDR-adjusted p <0.05) differences between serum tyrosine (sTYR) threshold groups in patients on nitisinone (visits 4-6 samples combined). Arrows indicate direction of change versus <701 µmol/L sTYR group; red arrows indicate increased abundance and blue arrows indicate decreased abundance. All data are from patients on nitisinone treatment. Serum tyrosine groups were assigned from a previous quantitative analysis of the same sample set[1].

Metabolite	*p* value (adjusted)	sTYR group (µmol/L) pairwise fold change comparisons		
		<701 *vs* 701-900	<701 *vs* 900-1100	<701 *vs* >1100
Phenylalanine *N*-acetylcysteine	0.00247	1.8 ↑	1.8 ↑	2.2 ↑
O-Methyl-phenylalanine	0.00025	2.1 ↑	2.3 ↑	3.5 ↑
Phenylpyruvic acid	0.00019	2.1 ↑	2.1↑	2.6 ↑
Phenyllactic acid	0.00019	2.1 ↑	2.0 ↑	2.8 ↑
Phenylacetamide	0.00019	2.2 ↑	2.4 ↑	3.1 ↑
Tyrosine	0.00199	1.7 ↑	1.6 ↑	2.4 ↑
N-Acetyl-tyrosine	0.00019	2.3 ↑	2.4 ↑	3.6 ↑
Tyrosine sulfate	0.00242	1.5 ↑	1.5 ↑	2.1 ↑
Tyrosine glucuronide	0.00325	2.5 ↑	1.7 ↑	2.8 ↑
Tyramine	0.02602	1.8 ↓	1.0 -	2.1 ↑
HPPA	0.00325	1.9 ↑	1.9 ↑	2.1 ↑
HPPA-hydrate	0.01270	1.5 ↑	1.9 ↑	1.9 ↑
HPLA	0.00176	1.9 ↑	1.9 ↑	2.2 ↑
HPLA-glycine	0.00140	4.9 ↑	4.4 ↑	4.9 ↑
HPLA-sulfate	0.00046	2.3 ↑	2.0 ↑	2.2 ↑

[1] Ranganath, L.R.; Milan, A.M.; Hughes, A.; Davison, A.S.; Khedr, M.; Norman, B.P.; Bou-Gharios, G.; Gallagher, J.A.; Imrich, R.; Arnoux, J.B.; et al. Determinants of Tyrosinaemia during Nitisinone Therapy in Alkaptonuria. Sci. Rep. 2022, 12, 16083. https://doi: 10.1038/s41598-022-20424-z.

Table S4. Comparison of urine metabolites between patients in the treated group who developed tyrosinaemia-associated corneal dendritiform keratopathy (KP; N = 5) and those who did not (N = 48). Values are mean (±SD) peak area, 24-h creatinine normalised, pareto scaled and log_2 transformed. Visits 1, 4 and 6 refer to baseline, 24 and 48 months respectively. Patients in the treated group were on nitisinone at visits 4 and 6.

	Visit 1		Visit 4		Visit 6	
Metabolite	KP	No KP	KP	No KP	KP	No KP
Phenylalanine	0.09 (±0.48)	-0.17 (±1.2)	-1.09 (±2.78)	0.03 (±1.15)	-0.01 (±0.65)	0.06 (±0.99)
Phenylalanine hydrate	0.47 (±0)	-0.07 (±1.3)	-1.29 (±1.93)	-0.33 (±1.2)	0.21 (±0.76)	0.48 (±0.95)
Phenylalanine *N*-acetylcysteine	-0.21 (±0)	-0.26 (±0.34)	0.58 (±0.46)	0.47 (±1.91)	0.38 (±0.55)	0.78 (±1.1)
O-Methyl-phenylalanine	-0.29 (±0.51)	-0.47 (±1.02)	0.56 (±0.85)	0.63 (±0.71)	0.57 (±0.55)	0.79 (±0.66)
Phenylpyruvic acid	-0.45 (±0)	-0.47 (±0.17)	0.68 (±0.64)	0.95 (±0.56)	0.43 (±0.68)	1.11 (±0.42)
Phenyllactic acid	-1.34 (±2.95)	-0.31 (±0.84)	0.24 (±0.51)	0.44 (±0.85)	0.22 (±0.55)	0.83 (±0.58)
Phenylacetamide	-1.36 (±3.25)	-0.33 (±1.18)	0.14 (±1.3)	0.26 (±1.23)	0.47 (±0.46)	0.65 (±0.91)
Phenylacetylglutamine	-0.55 (±1.32)	-0.04 (±1.11)	0.1 (±0.65)	-0.01 (±1.05)	0.02 (±0.7)	0.3 (±0.9)
Tyrosine	-0.88 (±1.52)	-0.47 (±1.26)	0.59 (±0.42)	0.29 (±1.06)	0.29 (±0.4)	0.68 (±0.69)
N-Acetyl-tyrosine	-0.31 (±0.66)	-0.49 (±1.44)	0.45 (±0.84)	0.51 (±0.71)	0.57 (±0.55)	0.76 (±0.51)
Tyrosine sulfate	0.23 (±0.67)	-0.12 (±1.15)	-0.89 (±1.89)	0.09 (±1.13)	-0.75 (±1.74)	0.21 (±0.99)
Tyrosine glucuronide	-0.95 (±1.47)	-0.25 (±0.78)	0.23 (±1.58)	0.76 (±0.79)	0.61 (±0.49)	0.77 (±0.75)
Tyramine	0.28 (±0.21)	-0.27 (±1.16)	0.72 (±0.83)	0.6 (±1.03)	-0.37 (±0.68)	0.46 (±0.72)
HPPA	-1.17 (±1.42)	-0.54 (±0)	0.72 (±0.72)	1.09 (±0.35)	-0.29 (±2.32)	1.24 (±0.34)

HPPA-hydrate	0.23 (±0)	-0.31 (±1.25)	0.25 (±0.24)	0.16 (±1.01)	0.54 (±0.36)	0.68 (±0.69)
HPPA-sulfate	0.08 (±0.22)	-0.14 (±1.04)	0.2 (±0)	-0.07 (±1.48)	0.15 (±0.43)	0.48 (±1.08)
HPLA	-0.19 (±0)	-0.66 (±1.75)	0.61 (±0.45)	0.74 (±0.68)	0.43 (±0.58)	0.96 (±0.32)
HPLA-glycine	-0.56 (±0)	-0.56 (±0)	0.84 (±0.84)	1.12 (±0.7)	0.56 (±1.03)	1.1 (±0.69)
HPLA sulfate	-0.22 (±0)	-0.3 (±0.41)	-0.1 (±1.25)	0.43 (±1.33)	0.35 (±0.67)	0.9 (±0.55)
HGA	0.06 (±0.58)	0.11 (±0.4)	-2.08 (±4.44)	-0.07 (±0.07)	-1.36 (±3.44)	-0.14 (±0.42)
Acetyl-HGA	-0.19 (±1.21)	0.09 (±0.95)	-0.15 (±0.84)	0.04 (±0.81)	0.47 (±0.34)	-0.02 (±1.01)
HGA-glucuronide	0.26 (±0.46)	0.29 (±0.55)	-0.44 (±0.04)	-0.59 (±0.87)	-0.05 (±0.52)	-0.54 (±0.68)
HGA sulfate	0.55 (±0.37)	0.21 (±0.76)	-0.27 (±0.13)	-0.2 (±0.08)	0.16 (±0.52)	-0.2 (±0.14)
